# Modulatory Effects of Caffeine and Pentoxifylline on Aromatic Antibiotics: A Role for Hetero-Complex Formation

**DOI:** 10.3390/molecules26123628

**Published:** 2021-06-14

**Authors:** Anna Woziwodzka, Marta Krychowiak-Maśnicka, Grzegorz Gołuński, Anna Felberg, Agnieszka Borowik, Dariusz Wyrzykowski, Jacek Piosik

**Affiliations:** 1 Laboratory of Biophysics, Intercollegiate Faculty of Biotechnology University of Gdansk and Medical University of Gdansk, 80-307 Gdansk, Poland; grzegorz.golunski@ug.edu.pl (G.G.); anna.felberg@phdstud.ug.edu.pl (A.F.); agnieszka.borowik@phdstud.ug.edu.pl (A.B.); jacek.piosik@ug.edu.pl (J.P.); 2 Laboratory of Biologically Active Compounds, Intercollegiate Faculty of Biotechnology University of Gdansk and Medical University of Gdansk, 80-307 Gdansk, Poland; marta.krychowiak@ug.edu.pl; 3 Department of Inorganic Biological Chemistry, Faculty of Chemistry, University of Gdansk, 80-308 Gdansk, Poland; dariusz.wyrzykowski@ug.edu.pl

**Keywords:** antibacterial agent, biological activity, caffeine, ciprofloxacin, natural compounds, tetracycline

## Abstract

Antimicrobial resistance is a major healthcare threat globally. Xanthines, including caffeine and pentoxifylline, are attractive candidates for drug repurposing, given their well-established safety and pharmacological profiles. This study aimed to analyze potential interactions between xanthines and aromatic antibiotics (i.e., tetracycline and ciprofloxacin), and their impact on antibiotic antibacterial activity. UV-vis spectroscopy, statistical-thermodynamical modeling, and isothermal titration calorimetry were used to quantitatively evaluate xanthine-antibiotic interactions. The antibacterial profiles of xanthines, and xanthine-antibiotic mixtures, towards important human pathogens *Staphylococcus aureus*, *Enterococcus faecium*, *Escherichia coli*, *Acinetobacter baumannii*, *Klebsiella pneumoniae*, and *Enterobacter cloacae* were examined. Caffeine and pentoxifylline directly interact with ciprofloxacin and tetracycline, with neighborhood association constant values of 15.8–45.6 M^−1^ and enthalpy change values up to −4 kJ·M^−1^. Caffeine, used in mixtures with tested antibiotics, enhanced their antibacterial activity in most pathogens tested. However, antagonistic effects of caffeine were also observed, but only with ciprofloxacin toward Gram-positive pathogens. Xanthines interact with aromatic antibiotics at the molecular and in vitro antibacterial activity level. Given considerable exposure to caffeine and pentoxifylline, these interactions might be relevant for the effectiveness of antibacterial pharmacotherapy, and may help to identify optimal treatment regimens in the era of multidrug resistance.

## 1. Introduction

Increasing antimicrobial resistance (AMR) is a major healthcare threat globally. According to recent estimates in the European Union, AMR contributes to more than 670,000 infections and 33,000 deaths annually [1]. Among drug-resistant bacteria, third-generation cephalosporin-resistant *Escherichia coli* and methicillin-resistant *Staphylococcus aureus* represent the most frequent and deadly causes of infection [1]. The burden of infections due to AMR has increased since 2007, and currently exceeds that of tuberculosis, influenza, and HIV infections combined [1]. In the United States, the extent of AMR is similar, with nearly three million antibiotic-resistant infections and 35,000 deaths reported each year [2].

Most infections with antibiotic-resistant bacteria are associated with healthcare institutions [1]. Therefore, the most vulnerable individuals hospitalized, due to chronic conditions, anticancer treatment, organ transplant, etc., are at the highest risk of acquiring difficult to treat infections caused by antibiotic-resistant bacteria [2]. The ongoing COVID-19 pandemic, as well as the past H1N1 influenza outbreak, show that, despite preventive antibiotic treatment [3,4], hospitalized patients are prone to developing secondary bacterial infections, which may significantly worsen their prognosis [4,5,6]. Indeed, bacterial co-infections were reported as a negative prognostic factor in the 2009 influenza A H1N1 pandemic, during which one in four patients suffered secondary bacterial infections [7]. This further emphasizes the urgent need to provide the healthcare system with a wide range of effective broad-spectrum antimicrobials.

The pipeline for new antimicrobials, especially those to treat multidrug-resistant bacteria, is narrow [8]. Remarkably, between the 1960s and 2000s, no novel class of antibiotics entered the market [9]. Recent advances in drug discovery, especially those focusing on natural products rather than synthetic compounds, combined with a growing body of initiatives aimed at promoting antimicrobial research, led to the discovery of promising new candidates with diverse modes of action [10]. However, derivatives of well-established antibiotic classes prevailed in the 2018 clinical pipeline of antibacterial agents, and most candidates showed only a limited level of innovation [11]. The analysis highlighted a particular demand for new compounds with no pre-existing cross-resistance to treat infections caused by Gram-negative bacteria [11].

One of the approaches to overcome drug resistance, or to reactivate the target for the antibiotic, is to use additional compounds, such as natural biologically active substances, or even existing drugs, as adjuvants of antibiotics. As evidence, amphotericin C was approved to treat visceral leishmaniasis [12] and doxycycline for chemoprophylaxis and malaria treatment [13]. More recently, the anthelmintic niclosamide was reported as a promising antibacterial agent [14]. The co-administration of β-lactam antibiotics with β-lactamase inhibitors, such as clavulanic acid, is another successful synergistic strategy [15]. Furthermore, the antidepressant sertraline was recently evaluated in late clinical trials as an adjuvant for antifungal treatment, and the antiprotozoal pentamidine proved effective in sensitizing Gram-negative bacteria to antibiotics and overcoming colistin resistance [16].

Xanthines, including caffeine and pentoxifylline, have well-established safety and pharmacological profiles, making them attractive candidates as drug adjuvants. Caffeine, a component of popular beverages, foods, dietary supplements, and drugs, is the most abundantly consumed pharmacologically-active substance worldwide. The estimated mean daily caffeine intake is 165 mg, and approximately 105 mg is related to drinking coffee [17]. The average annual consumption of caffeine-containing beverages (mostly coffee and carbonated soft drinks) is 348 L per person in North America and 200 L per person in Europe [18]. As a drug, it is used to treat apnea of prematurity by reducing bronchopulmonary dysplasia [19]. It is also used in combination with analgesics as a pain reliever [20], and in the treatment of hypersomnia [21]. Meanwhile, pentoxifylline is commonly used in pharmacotherapy to treat vascular diseases, including intermittent claudication, venous leg ulcers [22], and heart failure [23,24] thanks to its anti-inflammatory and rheological properties.

Caffeine and pentoxifylline were also shown to diminish the activity of a broad range of small aromatic compounds (e.g., model mutagens [25], anticancer drugs [26,27], neurotoxins [28], or foodborne carcinogens [29,30]) through their sequestration in transient non-covalent complexes and subsequent lowering of bioavailability. Although caffeine and pentoxifylline are considered safe, even at relatively high doses (up to 400 mg/day for caffeine and a typical dose of 1200 mg/day for pentoxifylline) [23,31], and their pharmacological activity is well-established, their antibacterial properties and modulatory effects on clinically-used antibiotics remain unclear.

This study aimed to investigate whether two aromatic-containing antibiotics (tetracycline and ciprofloxacin) interact non-covalently with caffeine and pentoxifylline. As such sequestration might affect biological activity of antibiotics captured in the hetero-complexes, we also evaluated the impact of xanthines on the in vitro activity of antibiotics toward a panel of seven Gram-positive and Gram-negative human pathogens. Given both considerable exposure to xanthines (e.g., as food constituents and/or drugs) and lack of effective treatment options for several emerging bacterial infections, knowledge on possible modulatory action of xanthines, toward antibiotics commonly used in clinical practice, appears relevant.

## 2. Results

### 2.1. Antibiotic-Xanthine Interactions: Spectrophotometric and Statistical Modelling Analysis

**Figure 1:**
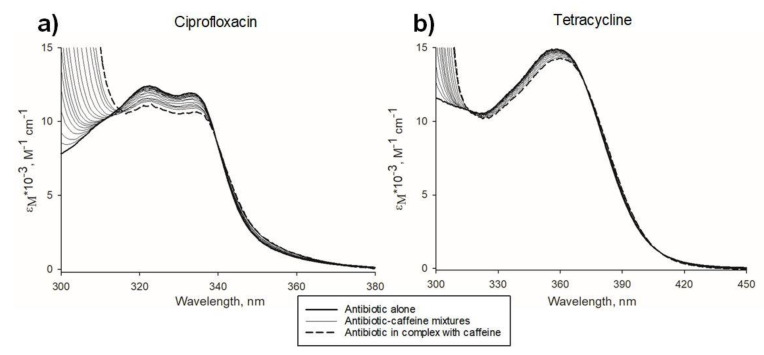
Spectrophotometric titrations of antibiotics with caffeine. (**a**), absorption spectra (in the form of molar extinction coefficient ε_M_) of ciprofloxacin (initial concentration, 60.3 µM) titrated with caffeine (concentration range, 0.3–40.6 mM); (**b**), absorption spectra (in the form of molar extinction coefficient ε_M_) of tetracycline (initial concentration, 43.2 µM) titrated with caffeine (concentration range, 0.3–21.5 mM). Spectra of an antibiotic in its free form are marked in bold. The spectra of antibiotics complexed with caffeine (calculated by extrapolation of mixture spectra to concentration ratio of antibiotic:caffeine → 0) are marked as dashed lines. For detailed composition of each tetracycline-caffeine mixture measured during spectrophotometric titration, see Table 1.

To investigate the direct interactions between antibiotics and xanthines in aqueous solutions, we performed UV-vis spectroscopic titrations of the antibiotic solution with caffeine or pentoxifylline. All absorption spectra were analyzed at wavelengths >320 nm, for which xanthine absorption is negligible. Absorptions spectra normalized to the concentration of the absorbing ligand (antibiotic) for antibiotic-caffeine titrations are shown in Figure 1.

In the analyzed range of concentrations, tetracycline and ciprofloxacin are present as monomers (no dimerization or higher-order aggregation was recorded). Observed spectral changes (represented most prominently by the hypochromic shift) can therefore be attributed to a new component that emerged upon the addition of xanthine solution (i.e., the antibiotic complexed with xanthine). The presence of an isosbestic point at 372 nm for tetracycline-caffeine mixtures (and at 339.5 nm for ciprofloxacin-caffeine mixtures) indicates that only two absorbing entities are present in the mixture (i.e., antibiotic monomer and antibiotic in hetero-complex with xanthine). Corresponding spectral changes were observed for tetracycline-pentoxifylline and ciprofloxacin-pentoxifylline mixtures, thus demonstrating ligand hetero-complexation.

**Figure 2:**
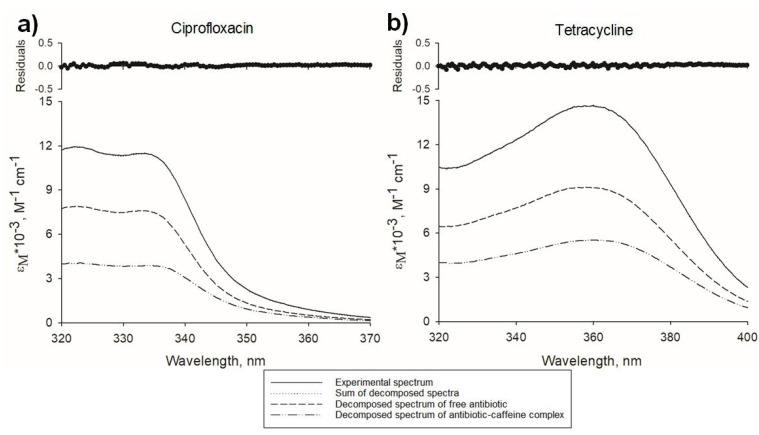
Examples of two-component decomposition of antibiotic-caffeine mixture spectra. (**a**), decomposition of mixture spectrum containing ciprofloxacin (55.1 µM) and caffeine (10.6 mM), molar fraction of free ciprofloxacin = 0.64; (**b**), decomposition of mixture spectrum containing tetracycline (40.4 µM) and caffeine (7.78 mM), molar fraction of free tetracycline = 0.61. Solid lines represent experimental spectra, dotted lines—sum of calculated decomposed spectra, dashed lines—calculated spectra of free antibiotics, dashed-dotted lines—calculated spectra of antibiotics complexed with caffeine. Top panels show residuals of experimental and the sum of calculated decomposed spectra.

Once spectra of the antibiotic, complexed with xanthine, were calculated, based on the law of spectra additivity, it was possible to estimate the molar fraction of free and complexed antibiotic in each mixture during spectrophotometric titration. Examples of such two-component spectra decomposition for selected ciprofloxacin-caffeine and tetracycline-caffeine mixtures are shown in Figure 2.

**Figure 3:**
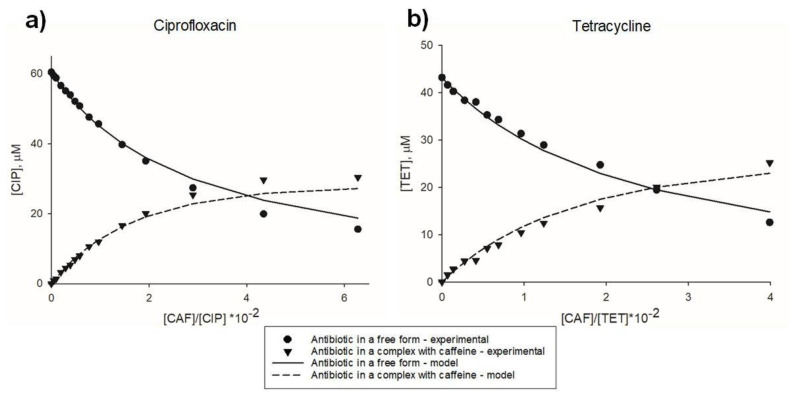
Comparison of experimental and theoretical concentrations in antibiotic-xanthine mixtures analyzed spectrophotometrically. (**a**), ciprofloxacin-caffeine interactions; (**b**), tetracycline-caffeine interactions. Points represent concentrations of antibiotic in a free form (circles) and in a complex with caffeine (triangles), calculated with two-component spectra decomposition. Lines represent concentrations of an antibiotic in a free form (solid line) and in a complex with caffeine (dashed line), calculated using statistical-thermodynamical model of mixed aggregation [32] (with K_AC_ ± standard error values 24.71 M^−1^ ± 0.89 M^−1^ for ciprofloxacin-caffeine interaction and 45.6 M^−1^ ± 2.5 M^−1^ for tetracycline-caffeine interaction). CAF, caffeine, CIP, ciprofloxacin. TET, tetracycline.

To quantitatively analyze the interactions, and calculate interaction constants, the statistical-thermodynamical model described by Zdunek et al. [32] was applied. The model assumes infinite aggregation of one type of ligand (xanthine), and limited aggregation of the heterocomplex formation of the other type of ligand (antibiotic). Experimental and theoretical concentrations of all components present in a mixture of tetracycline and caffeine are given in Table 1. Values of the neighborhood association constant K_AC_ for antibiotic-xanthine interactions, calculated with the model, were in the range of 10 M^−1^ (Table 2). The fit of the model to the experimental data for ciprofloxacin-caffeine and tetracycline-caffeine interactions is shown in Figure 3.

Upon the addition of caffeine, concentrations of antibiotics present in a free form markedly decreased. A ~250–300-fold excess of caffeine molecules, over antibiotic molecules, was needed to sequester half of the antibiotic molecules in hetero-complexes.

### 2.2. Thermal Effects of Antibiotic-Xanthine Interactions

**Figure 4:**
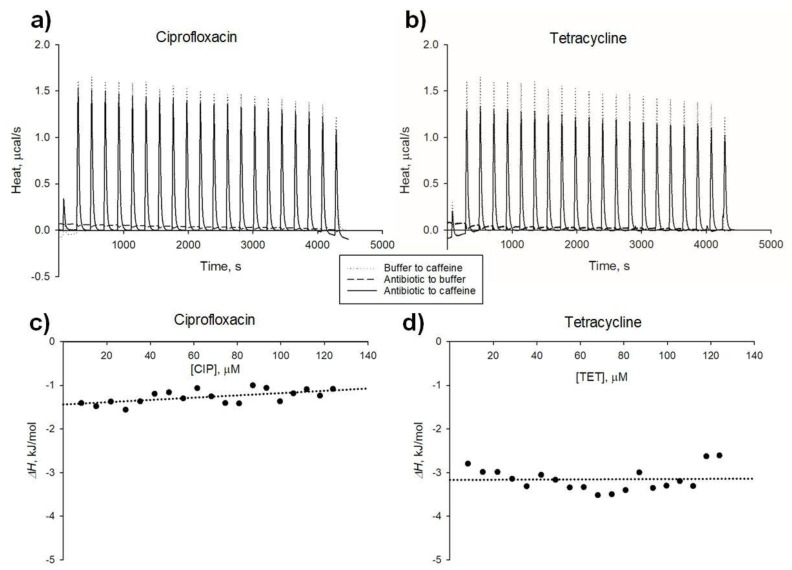
Thermal effects of antibiotic-caffeine complex formation—analysis with isothermal titration calorimetry. (**a,b**), thermograms for analysis of ciprofloxacin-caffeine (**a**) and tetracycline-caffeine (**b**) interactions; solid line, titration of caffeine with antibiotic; dotted line, titration of caffeine with buffer; dashed line, titration of buffer with antibiotic; (**c,d**), determination of enthalpy change (Δ*H*) values for ciprofloxacin-caffeine (**c**) and tetracycline-caffeine (**d**) interactions. Points correspond to net heat of antibiotic-xanthine interaction (corrected for heats of buffer-to-xanthine and antibiotic-to-buffer titrations, see Figure S1) per mole of titrant added. CIP, ciprofloxacin. TET, tetracycline.

To further characterize the interactions of tetracycline and ciprofloxacin with the xanthines, ITC measurements were performed (Figure 4). Peaks from thermograms associated with titrations of antibiotic (or buffer) with xanthine (or buffer) were integrated to estimate the heat of interaction. The net heat effect of antibiotic-xanthine was calculated as the heat effect of antibiotic-xanthine titration corrected for the heat of dilution of the antibiotic and the xanthine (recorded in control buffer titrations, Supplementary Figure S1). To estimate the enthalpy change (Δ*H*) of antibiotic-xanthine hetero-aggregation, the net heat of interaction per mole of titrant added was extrapolated for antibiotic concentration → zero (as shown in Figure 4c,d). Δ*H* values for antibiotic-xanthine interaction are listed in Table 2.

### 2.3. Antibacterial Activity of Caffeine and Pentoxifylline

To evaluate the modulatory effects of the xanthines on selected antibiotics, the antibacterial activity of caffeine and pentoxifylline alone was first determined. MIC values for caffeine and pentoxifylline were evaluated for a series of Gram-positive and Gram-negative pathogens using the broth microdilution procedure (Table 3). Caffeine was not active against *S. aureus*, *E. faecium*, and *P. aeruginosa* up to 16 mg/mL, and demonstrated limited antibacterial activity against Gram-negative *E. coli*, *A. baumannii*, *K. pneumoniae*, and *E. cloacae* with MIC values of 4–8 mg/mL. Pentoxifylline showed no antibacterial activity at concentrations up to 16 mg/mL.

### 2.4. Modulation of Antibiotic Activity by Caffeine and Pentoxifylline

To investigate the possible impact of caffeine and pentoxifylline on the antibacterial activity of antibiotics, the inhibitory effects of xanthine-antibiotic mixtures on microbial growth were investigated, for a broad range of concentrations, using a checkerboard titration technique, and the corresponding MIC values were determined for each mixture.

**Figure 5:**
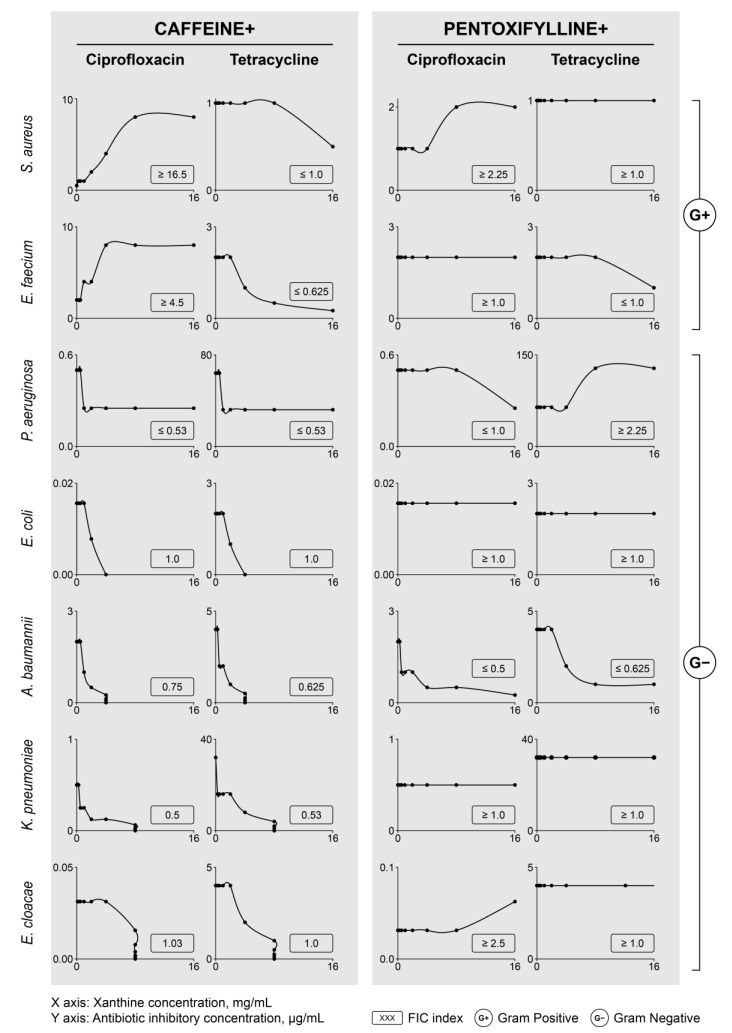
Dose-dependent modulation of antibiotics (ciprofloxacin and tetracycline) inhibitory potential by xanthines (caffeine and pentoxifylline) towards selected bacterial pathogens. Graphs represent isobolograms for each antibiotic-xanthine pair tested in concentration gradient of both compounds. Seven investigated pathogens are given as separate rows. Inhibitory concentrations given on Y axes correspond to minimal inhibitory concentration of tested antibiotics. FIC, Fractional Inhibitory Concentration Index, calculated for each tested antibiotic-xanthine combination according to Odds [33].

Figure 5 shows an overview of the concentration-dependent effects of the xanthines on the antibacterial activity of ciprofloxacin and tetracycline, expressed as isobolograms obtained for all analyzed pathogens and antibiotic-xanthine combinations. The modulatory effect of the xanthines is presented as the change in inhibitory concentration of an antibiotic for increasing xanthine concentration. Full-size isobolograms for all pathogens, and each antibiotic-xanthine combination, are given in Supplementary Figures S2–S8.

The profile of antibiotic activity modulation is distinct for caffeine and pentoxifylline. Caffeine potentiates the antibacterial activity of both ciprofloxacin and tetracycline in all Gram-negative pathogens evaluated, with mostly additive effects, as determined by FICI values (synergy was observed only for caffeine toward ciprofloxacin in *K. pneumoniae*). However, in Gram-positive bacteria, only tetracycline antibacterial activity increased upon caffeine addition, while ciprofloxacin activity was inhibited. Meanwhile, the effects of pentoxifylline on the antibacterial potential of ciprofloxacin and tetracycline were less pronounced than those of caffeine. Meaningful potentiation of antibiotic activity by pentoxifylline was observed only for *A. baumannii*. Slight inhibitory effects of pentoxifylline were reported for ciprofloxacin in *S. aureus* and *E. cloacae*, as well as for tetracycline in *P. aeruginosa*. The MIC values for antibiotics, alone and in combination, with the highest sub-inhibitory concentration of caffeine and pentoxifylline are listed in Table 4.

## 3. Discussion

We showed that the xanthines caffeine and pentoxifylline are capable of forming non-covalent hetero-complexes with two aromatic antibiotics, tetracycline and ciprofloxacin. This hetero-complexation leads to a substantial decrease in the free antibiotic concentration when the xanthine is at ~100-fold or more excess compared to the antibiotic. We also demonstrated that caffeine and pentoxifylline alter the antibacterial properties of tetracycline and ciprofloxacin; i.e., they potentiate or reduce their activity, depending on the bacterial species.

Being the vital component of the broadly available beverages with psychostimulatory properties (e.g., coffee or tea), caffeine is consumed on a daily basis. The single doses of xanthines, administered as a part of everyday diet (caffeine), or in pharmacotherapy (pentoxifylline), reach levels of up to 100–400 mg (for caffeine-containing beverages equivalent to up to 2–7 mg/mL) [34], and peak plasma concentrations of 1–10 mg/L [35,36]. Yet, knowledge on the antibacterial activity of xanthines, and their possible impact on antibiotic therapy, is limited. Previous studies showed that caffeine at concentrations of 2 mg/mL inhibited the growth of Enterobacteria, particularly *Serratia marcescens* and *E. cloacae* [37]. Likewise, the calculated IC50 value of caffeine against *Salmonella enterica* was 2.6 mg/mL, demonstrating that caffeine concentration in coffee extracts is sufficient to inhibit growth of this pathogen [37]. Similarly, caffeine at concentrations of ≥5 mg/mL inhibited the growth of the pathogenic *E. coli* O157 strain [38]. Finally, caffeine at a concentration of 4 mg/mL prevented growth of a wild-type *E. coli* strain, whereas knock-out mutants (particularly those lacking functions related to DNA repair) had increased caffeine sensitivity with growth inhibition present at 2.5 mg/mL or lower [39]. In our study, caffeine showed MIC values of 4–8 mg/mL against *E. coli*, *E. cloacae*, *K. pneumoniae*, and *A. baumannii*. Meanwhile, pentoxifylline was previously shown to lack antibacterial activity against *E. coli* with MIC values >1 mg/mL [40], and our study confirmed this finding, with no inhibitory effects observed against a panel of Gram-positive and Gram-negative pathogens at concentrations up to 16 mg/mL.

Prior studies showed that extracts of roasted coffee exhibited antibacterial activity against human pathogens like *S. aureus* or *Streptococcus mutans*; however, the intrinsic antibacterial activity of caffeine was weak [41]. Yet, the addition of alpha-dicarbonyl compounds to caffeine synergistically increased its antibacterial properties [41]. Indeed, Kang et al. described the synergistic effects of caffeine on the aminoglycoside antibiotics, kanamycin and neomycin, in *E. coli* [39]. The authors concluded that aminoglycosides generated damage to bacterial DNA bases, and the synergistic action of caffeine was attributed to slowing, and ultimately blocking, DNA replication. Unexpectedly, the opposite effects were observed with the fluoroquinolone, ciprofloxacin: caffeine suppressed its antimicrobial effects toward *E. coli* and *Bacillus anthracis*. No possible explanation of such caffeine action was given [39]. As an aromatic molecule, caffeine may directly interact with ciprofloxacin, thus decreasing its antibacterial activity, which could explain its contradictory effects on ciprofloxacin compared to kanamycin and neomycin. A similar pattern of caffeine action was described for two model nitrogen mustard mutagens: caffeine prevented cytotoxicity of an aromatic, heterocyclic quinacrine mustard, whereas no modulatory effect of caffeine was shown for the aliphatic mechlorethamine [42].

In our study, using UV-Vis spectroscopy, combined with statistical-thermodynamical modeling and ITC, we provided evidence for non-covalent complex formation between xanthines (caffeine and pentoxifylline) and two aromatic antibiotics, ciprofloxacin and tetracycline. The neighborhood association constants (*K_AC_*) and enthalpy changes (Δ*H*) were lower than those determined for xanthines and model mutagens, heterocyclic foodborne carcinogens, or anticancer drugs (*K_AC_* in 10 M^−1^ range; Δ*H* values 20–30 kJ M^−1^) [25,30,43]. This may be due to the restricted availability of aromatic rings within the antibiotic molecules, when compared with classic aromatic ligands, which are characterized by dominating conjugated planar aromatic and/or heterocyclic structures. Nevertheless, the interception of antibiotic molecules was effective for a xanthine:antibiotic molar ratio of ≥100. The concentrations of xanthines used in our checkerboard experiments were typically at least 1000-fold higher than the concentrations of antibiotics. Under such conditions, hetero-complexation at the molecular level should be relevant, with at least 75% of the antibiotic molecules sequestered, according to xanthine-antibiotic association constants (calculated based on UV-Vis spectroscopy measurements). Such sequestration could result in the reduction of antibiotic activity. However, our in vitro analysis of antibacterial activity showed the opposite: for most studied pathogens, caffeine enhanced, rather than diminished, effects of both tetracycline and ciprofloxacin. Inhibitory effects of xanthines were reported for ciprofloxacin only, and were restricted to Gram-positive pathogens (*S. aureus* and *E. faecium*).

The observed reduction of ciprofloxacin activity in the presence of xanthines is in line with reports by Kang et al., who showed antagonistic activity of caffeine toward ciprofloxacin in *E. coli* [39], and by Masadeh et al., who described the inhibitory effects of pentoxifylline toward ciprofloxacin in a panel of Gram-positive and Gram-negative pathogens, including *S. aureus*, *P. aeruginosa*, *K. pneumoniae*, and *A. baumannii* [44]. Our findings indicate that mixed stacking aggregate formation, between xanthines and antibiotics, only reduce antibiotic activity if the mechanism of action of the affected antibiotic depends on DNA binding (as is the case for ciprofloxacin but not tetracycline). This is in agreement with the inhibitory effects of xanthines against model mutagens, anticancer drugs, or food-derived carcinogens described previously, as all of these compounds exert their biological action, at least in part, through non-covalent (intercalation) or covalent (adduct formation) DNA binding [25,26,30].

In contrast to Gram-positive bacteria, caffeine potentiated the antibacterial activity of both tetracycline and ciprofloxacin in Gram-negative pathogens. It seems plausible that the attenuation of ciprofloxacin by caffeine, observed in Gram-positive bacteria, even if present in Gram-negative pathogens, is surpassed by another mechanism of caffeine action, which is specific for Gram-negative bacteria. It could be speculated that heterocomplexation of an antibiotic with xanthine increases antibiotic solubility and/or membrane permeability (as xanthines penetrate into membranes easily) [45], which could lead to increased antibacterial response. However, heterocomplexation of aromatic antibiotics was reported for both caffeine and pentoxifylline, and the strength of interaction was comparable for both xanthines, whereas prominent potentiation of antibacterial activity of ciprofloxacin and tetracycline was only observed for caffeine. Therefore, the potentiating effect of caffeine on antibiotic action is most likely dependent on the antibacterial activity of caffeine itself rather than its interplay with antibiotics, as the observed modulatory effects were, at best, additive. Indeed, the potentiation of tetracycline and ciprofloxacin activity was only observed in *P. aeruginosa* caffeine concentrations of >1 mg/mL, although the bacteria were resistant to caffeine alone at concentrations up to 16 mg/mL. In contrast, pentoxifylline lacked antibacterial activity at concentrations up to 16 mg/mL, which likely explains its lack of influence on antibiotic activity for most studied organisms.

Overall, the activity of caffeine, both alone and in combination with ciprofloxacin and tetracycline, towards Gram-negative pathogens appears promising. The elucidation of its mechanism of action is appealing, especially considering there is a shortage of effective pharmacological treatment options particularly for Gram-negative-associated infections [46]. In addition, further evaluation of the possible modulatory effects of xanthines on quinolones is warranted, as quinolones represent one of the most intensively explored class of antibiotics, and several new fourth-generation fluoroquinolones have recently entered the market or are in late phase drug development [47]. While the antibacterial activity of caffeine with MIC values ≥4 mg/mL is insufficient to act as a standalone antibiotic, in this study we present that caffeine is capable of potentiating the effects of two aromatic antibiotics commonly used in the clinical practice. In most Gram-negative pathogens investigated, antibiotic MIC values were halved in the caffeine concentrations of 0.5 mg/mL. The caffeine peak plasma concentration in humans is 1–2 µg/mL following a standard oral dose of 50 mg [48,49]. However, several-fold higher caffeine doses are considered safe (~400 mg per day [31]), and, in particular, populations (e.g., psychiatric patients) daily caffeine consumption exceeds 750 mg [48]. Our study shows potential benefit in use of caffeine as an adjuvant in antibacterial therapy, and it can thus serve as a pave for further research. This can include structural modifications of a caffeine molecule or co-formulation with another adjuvant, with an aim to enhance its antibiotic-potentiating effects, or its local rather than systemic administration, which might prove clinically valuable. The finding of molecular hetero-complex formation between caffeine and aromatic antibiotics can have practical implications as orally administered antibiotics might be potentially sequestered by caffeine in the gastrointestinal tract following consumption of caffeine-rich beverages. This might be particularly important when individual caffeine ingestion exceeds the average exposure, or when the drug is taken simultaneously with consumption of a caffeine-rich beverage. Impact of caffeine on drug absorption has been broadly described previously [50,51,52], and it seems plausible that the hetero-complex formation observed in our study can also contribute to this phenomenon.

In conclusion, xanthines, such as caffeine and pentoxifylline, interact with aromatic antibiotics at the molecular and in vitro antibacterial activity level. Given the considerable exposure to caffeine and pentoxifylline, these interactions might be relevant for the effectiveness of antibacterial pharmacotherapy. According to our findings, caffeine potentiates the antibacterial activity of selected antibiotics in Gram-negative pathogens. Therefore, as a compound with a well-established safety profile, caffeine and its potential to enhance antibacterial action of known antibiotics may be worth further investigation when searching for optimal treatment regimens in the era of multidrug resistance.

## 4. Materials and Methods

### 4.1. Materials

**Figure 6:**
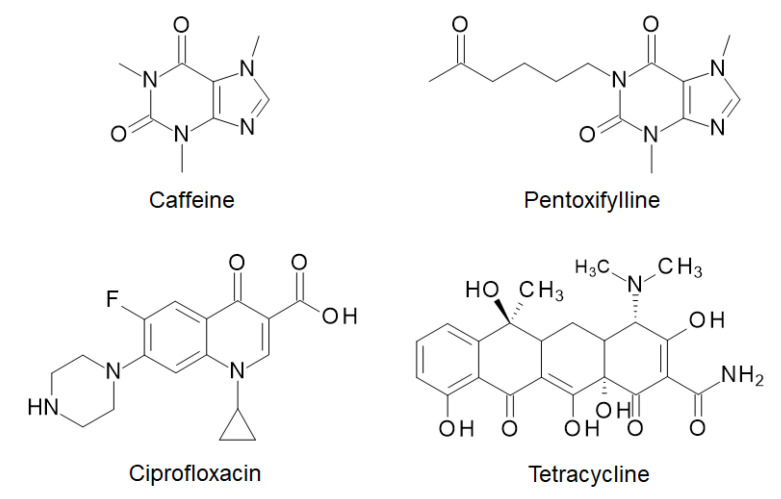
Chemical structures of studied compounds. Top, xanthines: caffeine and pentoxifylline; bottom, antibiotics: ciprofloxacin and tetracycline.

All chemicals, including xanthines caffeine (1,2,3-trimethylxanthine) and pentoxifylline (3,7-dimethyl-1-(5-oxohexyl)xanthine), and antibiotics tetracycline hydrochloride and ciprofloxacin hydrochloride, were purchased from Sigma-Aldrich (St. Louis, MO, USA). Structures of the above mentioned compounds are shown in Figure 6. A 0.1 M sodium phosphate buffer (pH 6.8), containing Na_2_HPO_4_ and NaH_2_PO_4_ (purchased from Avantor Performance Materials, Gliwice, Poland) was used in UV-Vis spectroscopy and ITC measurements. The buffer was filtered through a 0.2 μm pore Millex Millipore filter and degassed before experiments. Caffeine and pentoxifylline stock solutions were prepared by dissolving their weight amounts in a sodium phosphate buffer (pH 6.8) or deionized water at concentrations of approximately 10^−1^ M, and stored at 4 °C. Antibiotic stock solutions were prepared by dissolving their weight amounts in a sodium phosphate buffer (pH 6.8), or deionized water immediately before the experiments. The concentrations of antibiotic solutions were assessed by UV-Vis spectroscopy using determined molar absorption coefficients (ε_λ_), ε_358_ = 14,900 M^−1^ cm^−1^ and ε_322_ = 12,380 M^−1^ cm^−1^ for tetracycline and ciprofloxacin, respectively.

### 4.2. UV-Vis Spectroscopy Measurements

The 2 mL aliquots containing the antibiotic were placed in a quartz cuvette (1 cm light path) and titrated with 5–150 μL of caffeine or pentoxifylline solution. Example of changes in concentrations of all mixture constituents during titration are presented in Table 1. The absorption spectra of each mixture were measured using a Beckman DU 650 or a Jena Analytic Specord 50 Plus spectrophotometer (equipped with a water bath or a Peltier thermostat, respectively) at 0.5 nm intervals, and stored in a digital form. All measurements were done in a 0.1 M sodium phosphate buffer (pH 6.8) at 25 °C (±0.1 °C). After each titration, the solution was gently mixed and the cuvette placed in a thermostated holder for approximately 5 min to reach the target temperature. Absorption spectra are given in the form of molar absorption coefficient (ε_λ_, M^−1^ cm^−1^).

### 4.3. Quantitative Analysis of Antibiotic-Xanthine Interactions

To reflect changes only in a structure of antibiotics, all the UV-Vis spectra were analyzed in the range of wavelengths above 320 nm, for which light absorption of xanthines is negligible. The spectrum of the antibiotic in complex with the xanthine was calculated by extrapolation of molar extinction coefficient (for each wavelength) to *C_TA_*/*C_TC_* → 0 (where *C_TA_* and *C_TC_* are the total concentrations of the antibiotic and the xanthine, respectively). The spectra of mixtures containing the antibiotic and the xanthine were decomposed into a weighted sum of components (antibiotic in its free form and antibiotic in hetero-complexed with xanthine) by non-linear regression analysis. This allowed estimation of the concentration of free antibiotic and antibiotic complexed with xanthine for all mixtures analyzed spectroscopically.

### 4.4. Calculations with Statistical-Thermodynamical Model

Mixed association constant values (*K_AC_*) for antibiotic-caffeine and antibiotic-pentoxifylline complexation along with the concentrations of all mixture components were determined with statistical thermodynamics of mixed aggregation based on the Zdunek et al. model [32]. The model describes interactions in two-component ligand-xanthine mixtures, where one component, *C* (xanthine) is capable of both homo- and hetero-complexation, and the other, *A* (in this analysis the antibiotic), is only capable of hetero-complexation with xanthine. To determine neighborhood *K_AC_* (hetero-neighborhood) and *K_CC_* (homo-neighborhood) equilibrium constants with the model, a weight function for each oligomer needs to be calculated. *K_CC_* equilibrium constant values were determined using constant values of caffeine homo-complexation reported previously [53]. The equations used to calculate concentrations of each component in every form in the antibiotic-xanthine mixture are listed below:

(1) $$C_{TA}=C_{A}{\left[\frac{1-C_{C}(K_{CC}-K_{AC})}{1-C_{C}(K_{CC}+K_{AC}^{2}C_{A})}\right]}^{2}$$

(2) $$C_{TC}=C_{A}{\left[\frac{1+K_{AC}C_{A}}{1-C_{C}(K_{CC}+K_{AC}^{2}C_{A})}\right]}^{2}$$

(3) $$C_{AC}=2K_{AC}C_{A}C_{C}(1+K_{AC}C_{A})\frac{1-C_{C}(K_{CC}-K_{AC})}{{\left[1-C_{C}(K_{CC}+K_{AC}^{2}C_{A})\right]}^{2}}$$

(4) $$C_{CC}=K_{CC}{\left[\frac{C_{C}(1+K_{AC}C_{A})}{1-C_{C}(K_{CC}+K_{AC}^{2}C_{A})}\right]}^{2}$$

where *C_TA_* and *C_TC_* are total concentrations of antibiotic and xanthine, respectively, *C_A_* and *C_C_* are concentrations of free antibiotic and xanthine molecules, respectively, *C_AC_* is the concentration of antibiotic-xanthine hetero-neighborhoods, while *C_CC_* is the concentration of xanthine homo-neighborhoods. The calculations were performed using SigmaPlot 11 (Systat Software, Inc., San Jose, CA, USA), Microsoft Office Excel (Microsoft), and Mathcad Prime 6 (Parametric Technology Corporation, Boston, MA, USA) software.

### 4.5. Isothermal Titration Calorimetry (ITC)

All isothermal titration calorimetry (ITC) experiments were conducted in a 0.1 M sodium phosphate buffer (pH 6.8) at 25 °C using an AutoITC isothermal titration calorimeter (MicroCal, Malvern, UK), with 1.4491 mL of sample and reference cells. The cell containing deionized water was used as the reference. All solutions were degassed before titrations. The experiment consisted of injecting 10.02 μL (20 injections, 2 μL for the first injection only) of buffer solution of the appropriate antibiotic (1 mM) into the reaction cell initially containing xanthine in the buffer solution (15 mM). The titrant was injected in 5 min intervals to ensure that the titration peak returns to the baseline prior to the next injection. Each injection lasted 20 s. Background titrations were run using identical titrant with the pure buffer solution placed in the sample cell. To account for the heat of dilution, the result of a background titration was subtracted from each experimental titration. To achieve a homogeneous mixing in the cell, the stirrer speed was established at 300 rpm. To remove the effect of titrant diffusion across the syringe tip during the equilibration process, an initial 2 μL injection was removed from each data set before analysis. The data, specifically the heat normalized per mole of injectant, were processed with Origin 7 software from MicroCal.

### 4.6. Antibacterial Assays

Cation-adjusted Mueller-Hinton broth (CA-MHB) for antimicrobial susceptibility testing by broth microdilution method was purchased from Beckton Dickinson (BD Difco™ BBL™). Following bacterial strains recommended for antibiotics testing were used in the study: Gram-*positive Staphylococcus aureus* ATCC 25923 and *Enterococcus faecalis* ATCC 19433; Gram-negative *Pseudomonas aeruginosa* ATCC 27853, *Escherichia coli* ATCC 25922, *Acinetobacter baumannii* ATCC 19606, *Klebsiella pneumoniae* ATCC 700603, and *Enterobacter cloacae* ATCC 700323.

Antimicrobial potential of tested agents was determined by broth microdilutions method according to CLSI guidelines [54] in sterile, polystyrene, flat bottom 96-well plates for non-adeherent cultures (NEST^®^). Minimal Inhibitory Concentration (MIC) of antibiotics and xanthines was defined as their lowest concentration at which no visible bacterial growth was observed after 24 h stationary incubation at 37 °C. The following gradients of compounds concentration, obtained by serial 2-fold dilutions of medium, were applied: from 128 to 0.015625 µg/mL for antibiotics, and from 16 to 1 mg/mL for xanthines. From thus prepared solutions, 100 µL aliquots were transferred into 96-well plates. Next, wells were inoculated with 10 µL aliquots of bacterial suspension containing approximately 1 × 10^7^ CFU/mL obtained from liquid cultures in CA-MHB medium (6 h, 37 °C, 150 rpm) diluted in fresh medium. Checkerboard titration method was used to evaluate interactions of antibiotics and xanthines by applying two-dimensional combination of their concentration gradients. For agents with antibacterial activity, i.e., when MIC value was shown, the following gradient was applied: 2 × MIC, 1 × MIC, 0.5 × MIC, 0.25 × MIC, 0.125 × MIC, 0.06 × MIC, and 0.03 × MIC. Agents without antibacterial potential (xanthines mainly) were applied starting from the highest tested concentration, i.e., 16 mg/mL, and the following concentrations were tested: 16, 8, 4, 2, 1, 0.5 and 0.25 mg/mL. Results were analyzed with two following methods: calculation of Fractional Inhibitory Concentration Index (FICI) for each tested combination (according to Odds) [33], and isobologram analysis [55]. FICI was applied for the combination of the lowest concentration of both agents inhibiting the growth of bacteria, and was calculated according to the following equation:

FICI = (MICA + B/MICA) + (MICB + A/MICB),(5)

where:

MICA + B—the lowest concentration of compound A in the presence of the lowest concentration of compound B at which inhibitory effect is observed,

MICA—MIC of compound A tested alone,

MICB + A—the lowest concentration of compound B in the presence of the lowest concentration of compound A at which inhibitory effect is observed,

MICB—MIC of compound B tested alone.

For xanthines without antimicrobial effect on particular bacterial pathogens (MIC >16 mg/mL), their highest used concentration, i.e., 16 mg/mL, was considered as equal to or lower than 0.5 × MIC. FICI values were used to characterize following types of interaction: (i) synergistic for FICI ≤ 0.5, (ii) additive for FICI between 0.5 and 2.0, (iii) antagonistic for FICI ≥ 4.0 [33]. All microbiological experiments were done at least as biological triplicates.

## Figures and Tables

**Table 1:** Concentrations of all components present in tetracycline-caffeine mixtures during spectrophotometric titration (experimental and calculated with Zdunek et al. model [32]).

Sample	*C_TC_*, mM	*C_TA_*, µM	*C_C_*, mM	*C_CC_*, mM	*C_AC_*, µM	*C’_A_*, µM	*C_A_*, µM	*X’_BA_*, µM	*X_BA_*, µM	*K_AC_*, M^−1^
0	0.00	43.18	0.00	0.000	0.00	43.18	43.18	0.00	0.00	-
1	0.30	43.07	0.29	0.001	1.14	41.58	41.94	1.50	1.13	60.50
2	0.59	42.97	0.58	0.004	2.24	40.25	40.76	2.72	2.21	56.75
3	1.18	42.76	1.14	0.015	4.29	38.35	38.58	4.41	4.18	48.26
4	1.76	42.55	1.68	0.033	6.18	38.00	36.59	4.55	5.96	33.81
5	2.33	42.34	2.21	0.058	7.93	35.26	34.78	7.08	7.56	42.24
6	2.90	42.14	2.71	0.089	9.55	34.29	33.13	7.85	9.01	38.70
7	4.02	41.74	3.68	0.167	12.45	31.33	30.22	10.41	11.52	40.10
8	5.12	41.34	4.59	0.265	14.95	28.91	27.74	12.43	13.60	40.41
9	7.78	40.38	6.64	0.583	19.92	24.72	22.92	15.66	17.46	38.71
10	10.32	39.47	8.42	0.982	23.54	19.44	19.44	20.03	20.03	45.57
11	15.06	37.76	11.41	1.942	28.24	12.59	14.80	25.17	22.96	55.84

*C_TC_*, total caffeine concentration; *C_TA_*, total tetracycline concentration; *C_C_*, caffeine monomer concentration; *C_CC_*, caffeine homoaggregate neighborhood concentration; *C_AC_*, tetracycline-caffeine heteroaggregates neighborhood concentration; *C’_A_*, tetracycline monomer concentration (determined spectrophotometrically); *C_A_*, tetracycline monomer concentration; *X’_BA_*, tetracycline in heteroaggregates with caffeine concentration (determined spectrophotometrically); *X_BA_*, tetracycline in heteroaggregates with caffeine concentration; *K_AC_*, tetracycline-caffeine neighborhood association constant. Mean *K_AC_* ± standard error, 45.6 M^−1^ ± 2.5 M^−1^.

**Table 2:** Determined thermodynamical parameters of antibiotic-xanthine hetero-complex formation.

Interaction	*K_AC_* (SE), M^−1^	Δ*H* (SE), kJ × mol^−1^
Tetracycline-caffeine	45.6 (2.5)	−3.17 (0.14)
Tetracycline-pentoxifylline	15.8 (0.6)	−4.00 (0.06)
Ciprofloxacin-caffeine	24.7 (0.9)	−1.44 (0.07)
Ciprofloxacin-pentoxifylline	18.4 (1.0)	−2.01 (0.06)

*K_AC_*, neighborhood association**** constant; Δ*H*, enthalpy change; SE, standard error.

**Table 3:** Antibacterial activity of xanthines: caffeine and pentoxifylline against selected Gram-positive and Gram-negative pathogens.

Pathogen	MIC (mg/mL)	
	Caffeine	Pentoxifylline
**Gram-positive**		
*Staphylococcus aureus* ATCC 25923	>16	>16
*Enterococcus faecium* ATCC 19433	>16	>16
**Gram-negative**		
*Pseudomonas aeruginosa* ATCC 27853	>16	>16
*Escherichia coli* ATCC 25922	4	>16
*Acinetobacter baumannii* ATCC 19606	4	>16
*Klebsiella pneumoniae* ATCC 700603	8	>16
*Enterobacter cloacae* ATCC 700323	8	>16

MIC, minimal inhibitory concentration.

**Table 4:** The influence of xanthines on antimicrobial activity of selected antibiotics.

	MIC_A_	MIC_A+caffeine_ (FICI)	MIC_A+pentoxifylline_ (FICI)
	[µg/mL]		
***Staphylococcus aureus*** **ATCC 25923**			
Ciprofloxacin	0.5–1	8 (≥16.5)	1 (≥2.25)
Tetracycline	1	0.5 (≤1.0)	1 (≥1.0)
***Enterococcus faecium*** **ATCC 19433**			
Ciprofloxacin	2	8 (≥4.5)	2 (≥1.0)
Tetracycline	2	0.25 (≤0.625)	1 (≤1.0)
***Pseudomonas aeruginosa*** **ATCC 27853**			
Ciprofloxacin	0.5	0.25 (≤0.53)	0.25 (≤1.0)
Tetracycline	64	32 (≤0.53)	128 (≥2.25)
***Escherichia coli*** **ATCC 25922**			
Ciprofloxacin	0.0156	0.078 (1.0)	0.0156 (≥1.0)
Tetracycline	2	1 (1.0)	2 (≥1.0)
***Acinetobacter baumannii*** **ATCC 19606**			
Ciprofloxacin	2	0.5 (0.75)	0.25 (≤0.5)
Tetracycline	4	1 (0.625)	1 (≤0.625)
***Klebsiella pneumoniae*** **ATCC 700603**			
Ciprofloxacin	0.5	0.125 (0.5)	0.5 (≥1.0)
Tetracycline	32	8 (0.53)	32 (≥1.0)
***Enterobacter cloacae*** **ATCC 700323**			
Ciprofloxacin	0.03125	0.03125 (1.03)	0.0625 (≥2.5)
Tetracycline	4	2 (1.0)	4 (≥1.0)

MIC, minimal inhibitory concentration of an antibiotic; A, antibiotic tested alone; A+caffeine, antibiotic tested with caffeine at the highest sub-inhibitory caffeine concentration (corresponding to MIC/2 or, when MIC >16 mg/mL, the highest tested concentration; MIC values specified in Table 3); A+pentoxifylline, antibiotic tested with pentoxifylline at the highest sub-inhibitory pentoxifylline concentration tested (MIC specified in Table 3). FICI, Fractional Inhibitory Concentration Index, calculated for each tested antibiotic-xanthine combination according to Odds [33].

## Data Availability

The data presented in this study are available on request from the corresponding author.

## Supplementary Materials

The following are available online. Supplementary Figure S1. Thermal effects of ciprofloxacin-caffeine (a) and tetracycline-caffeine (b) interactions; circles, titration of caffeine with buffer; squares, titration of caffeine with antibiotic; triangles, titration of buffer with antibiotic. The net heat of antibiotic-caffeine interaction, calculated as the difference between heat of antibiotic-caffeine titration and control (buffer) titrations, is marked with crosses. Supplementary Figures S2–S8. Modulation of ciprofloxacin and tetracycline antibacterial activity by xanthines (caffeine and pentoxifylline) in *Staphylococcus aureus* (Figure S2), *Enterococcus faecium* (Figure S3), *Pseudomonas aeruginosa* (Figure S4), *Escherichia coli* (Figure S5), *Acinetobacter baumannii* (Figure S6), *Klebsiella pneumoniae* (Figure S7), *Enterobacter cloacae* (Figure S8) using microbroth dilution assay and checkerboard methodology. Panel a, ciprofloxacin-caffeine mixtures; panel b, tetracycline-caffeine mixtures; panel b, ciprofloxacin-pentoxifylline mixtures; panel d, tetracycline-pentoxifylline mixtures. FIC, Fractional Inhibitory Concentration Index.

## Sample Availability

All compounds used in the study are commercially available (for provider, see Materials and Methods section).
