# Supplementary material for: Modulatory Effects of Caffeine and Pentoxifylline on Aromatic Antibiotics: A Role for Hetero-Complex Formation
Source: Molecules. 2021 Jun 14;26(12):3628. doi: 10.3390/molecules26123628 (PMC8231999; doi:10.3390/molecules26123628)
Supplement: Supplementary file 1 [file molecules-26-03628-s001.zip › molecules-1234160-supplementary.pdf]

Article

Modulatory effects of caffeine and pentoxifylline on aromatic antibiotics: a role for hetero-complex formation

Anna Woziwodzka [1,*], Marta Krychowiak-Maśnicka [2], Grzegorz Gołuński [1], Anna Felberg [1], Agnieszka Borowik [1], Dariusz Wyrzykowski [3], Jacek Piosik [1]

[1] Laboratory of Biophysics, Intercollegiate Faculty of Biotechnology University of Gdansk and Medical University of Gdansk, 80-307 Gdansk, Poland; grzegorz.golunski@ug.edu.pl (GG), anna.felberg@phdstud.ug.edu.pl (AF), agnieszka.borowik@gmail.com (AB), jacek.piosik@ug.edu.pl (JP)
[2] Laboratory of Biologically Active Compounds, Intercollegiate Faculty of Biotechnology University of Gdansk and Medical University of Gdansk, 80-307 Gdansk, Poland; ; marta.krychowiak@ug.edu.pl
[3] Department of Inorganic Biological Chemistry, Faculty of Chemistry, University of Gdansk, 80-308 Gdansk, Poland; dariusz.wyrzykowski@ug.edu.pl
* Correspondence: anna.woziwodzka@ug.edu.pl

Figure S1a

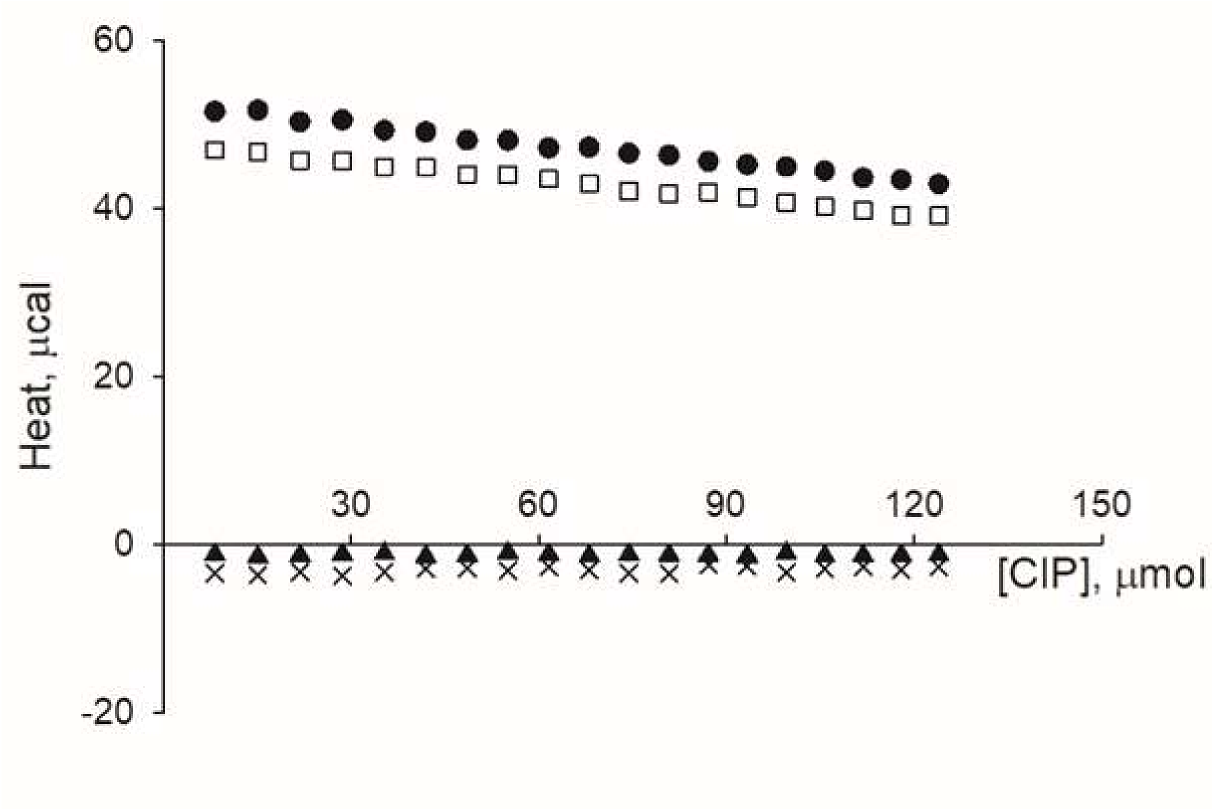

Figure S1b

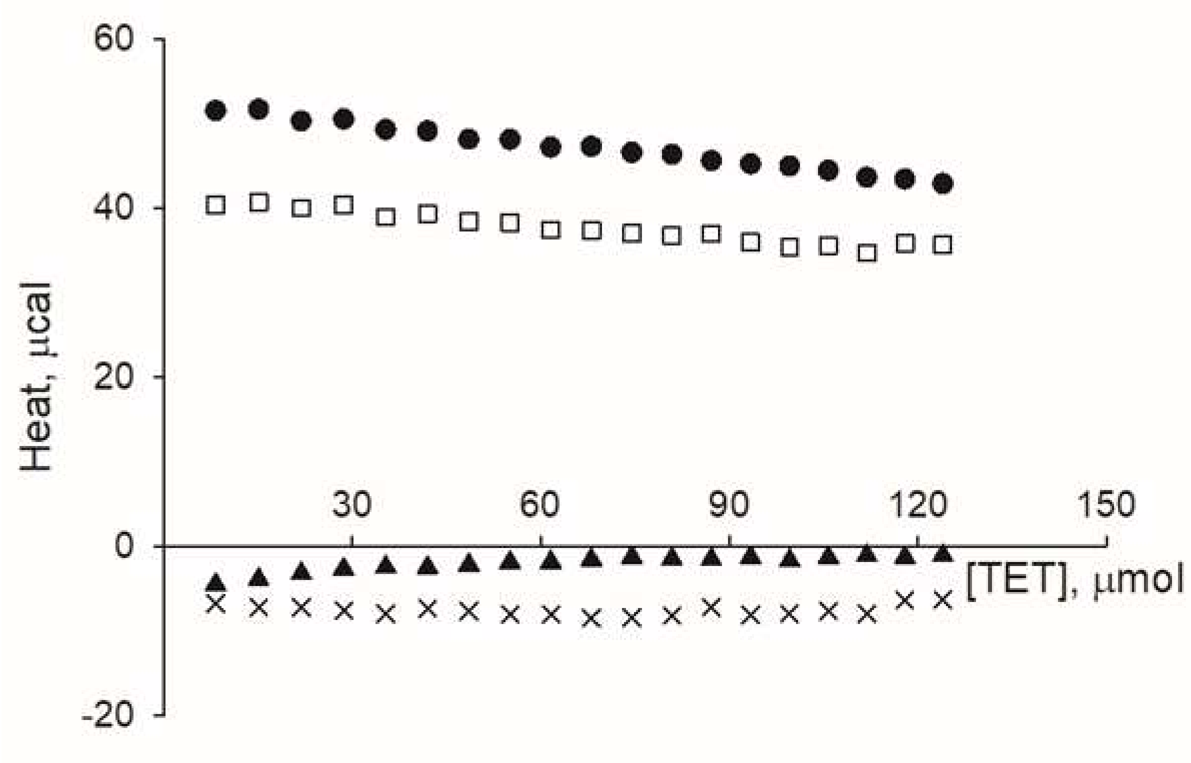

Supplementary Figure S1. Thermal effects of ciprofloxacin-caffeine (a) and tetracycline-caffeine (b) interactions; circles, titration of caffeine with buffer; squares, titration of caffeine with antibiotic; triangles, titration of buffer with antibiotic. The net heat of antibiotic-caffeine interaction, calculated as the difference between heat of antibiotic-caffeine titration and control (buffer) titrations, is marked with crosses. CIP, ciprofloxacin. TET, tetracycline

Figure S2a

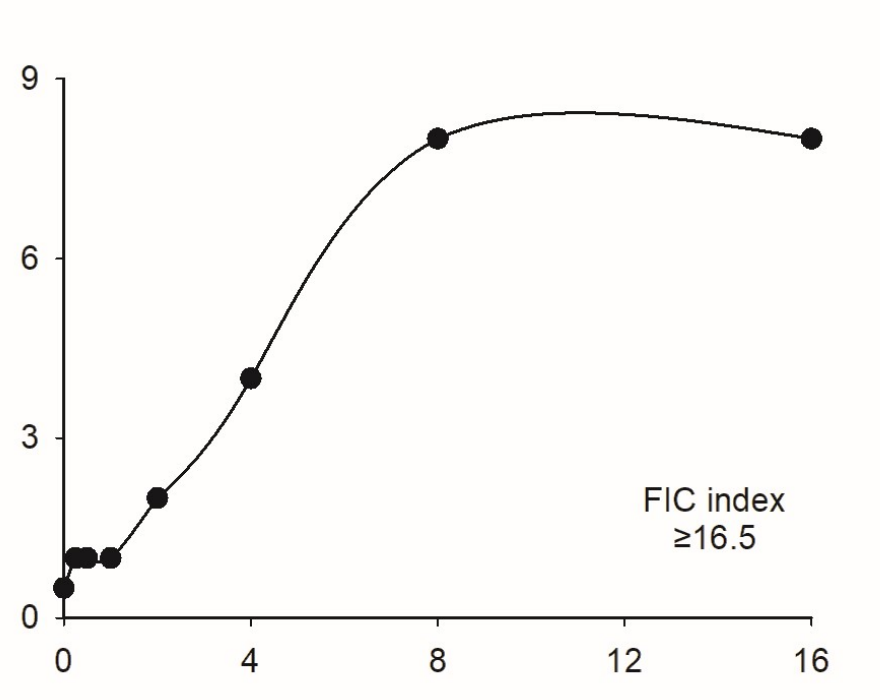

Figure S2b

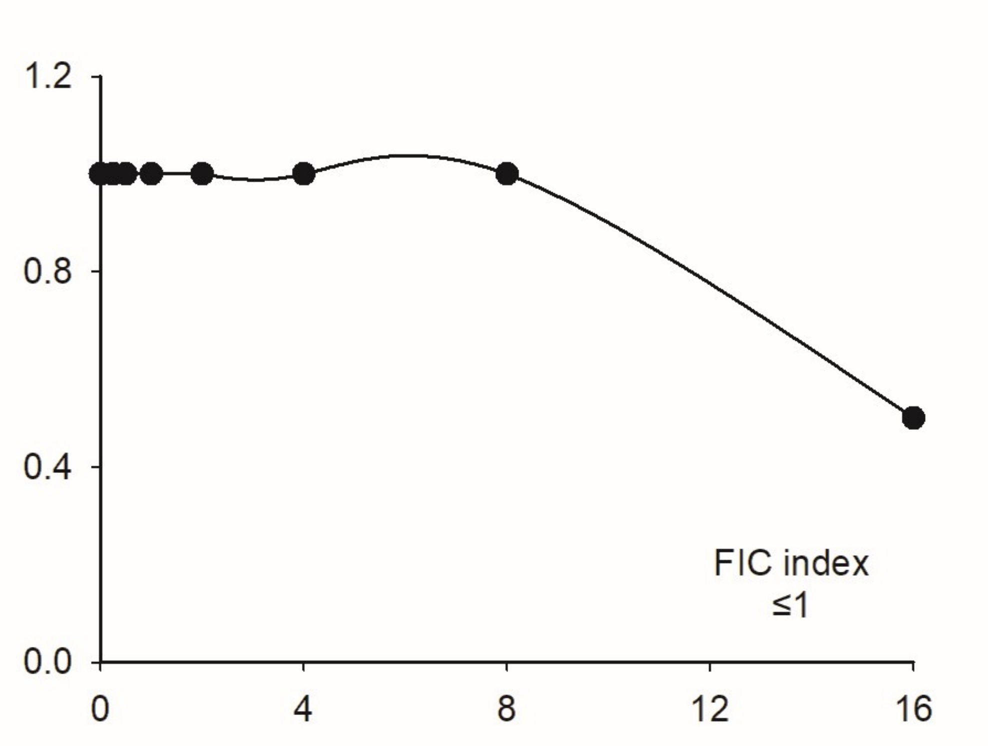

Figure S2c

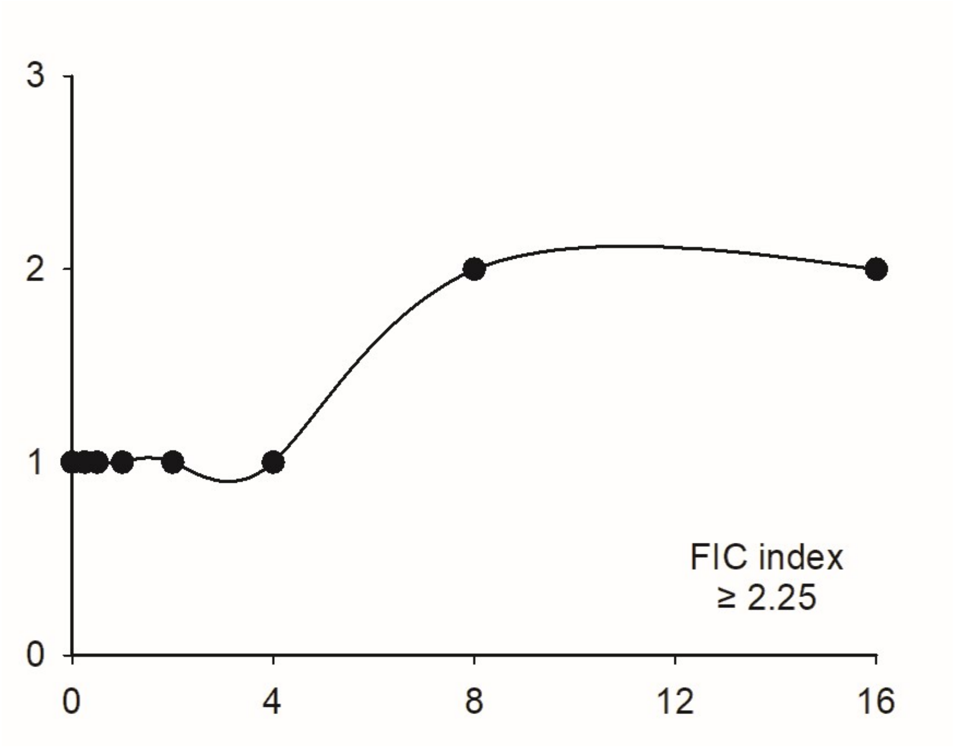

Figure S2d

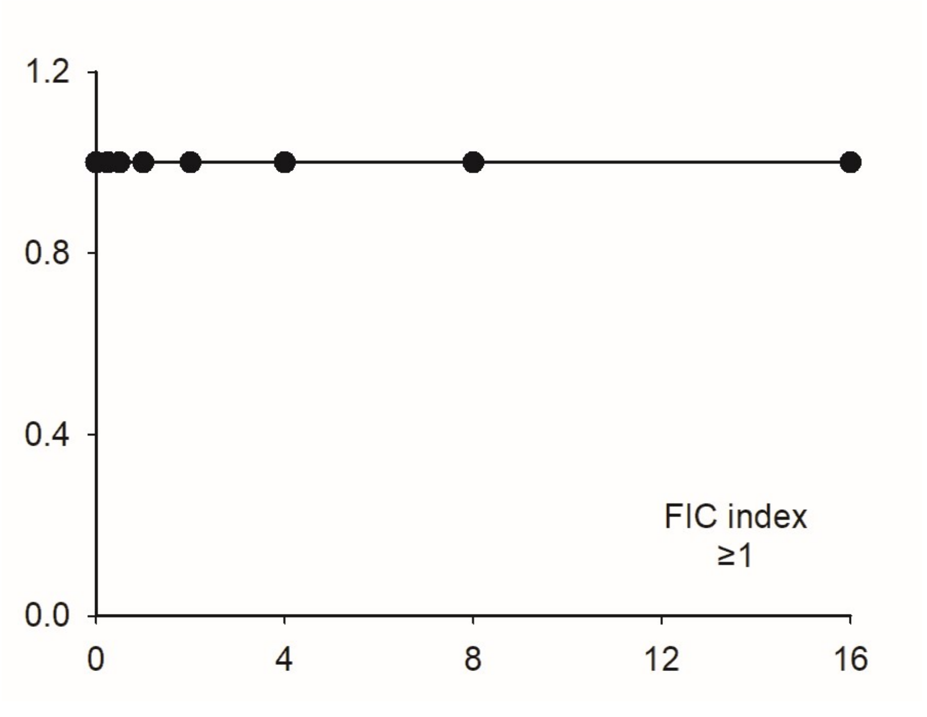

Supplementary Figure S2. Modulation of ciprofloxacin and tetracycline antibacterial activity by xanthines (caffeine and pentoxifylline) in *Staphylococcus aureus* using microbroth dilution assay and checkerboard methodology. (**a**), ciprofloxacin-caffeine mixtures; (**b**), tetracycline-caffeine mixtures; (**c**), ciprofloxacin-pentoxifylline mixtures; (**d**), tetracycline-pentoxifylline mixtures. FIC, Fractional Inhibitory Concentration Index calculated for each tested antibiotic-xanthine combination according to Odds [33]

Figure S3a

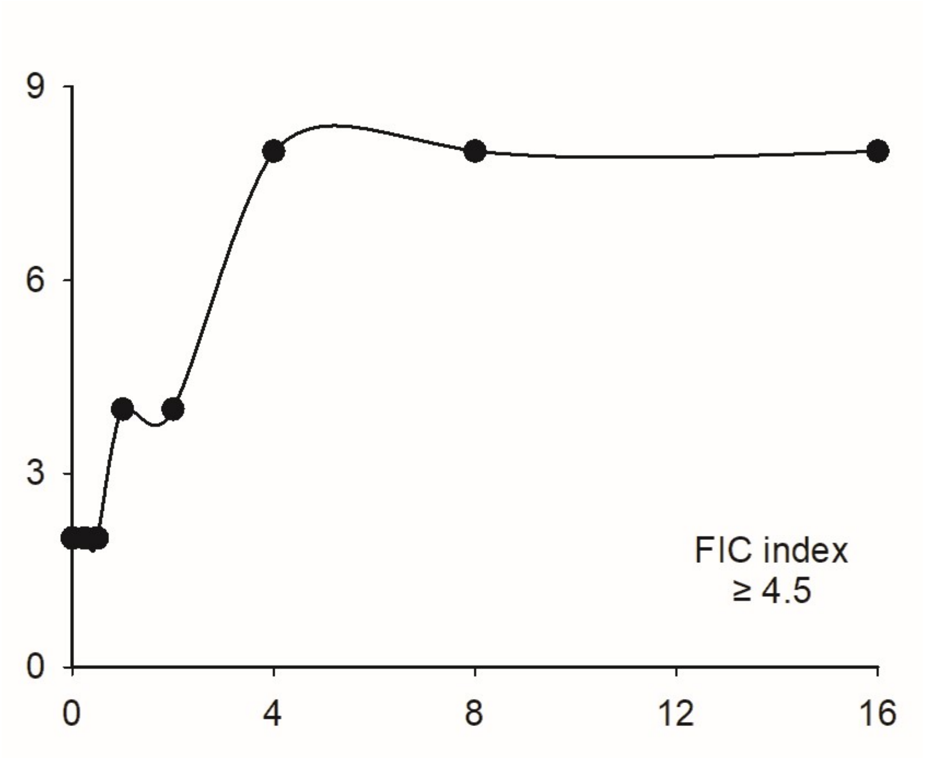

Figure S3b

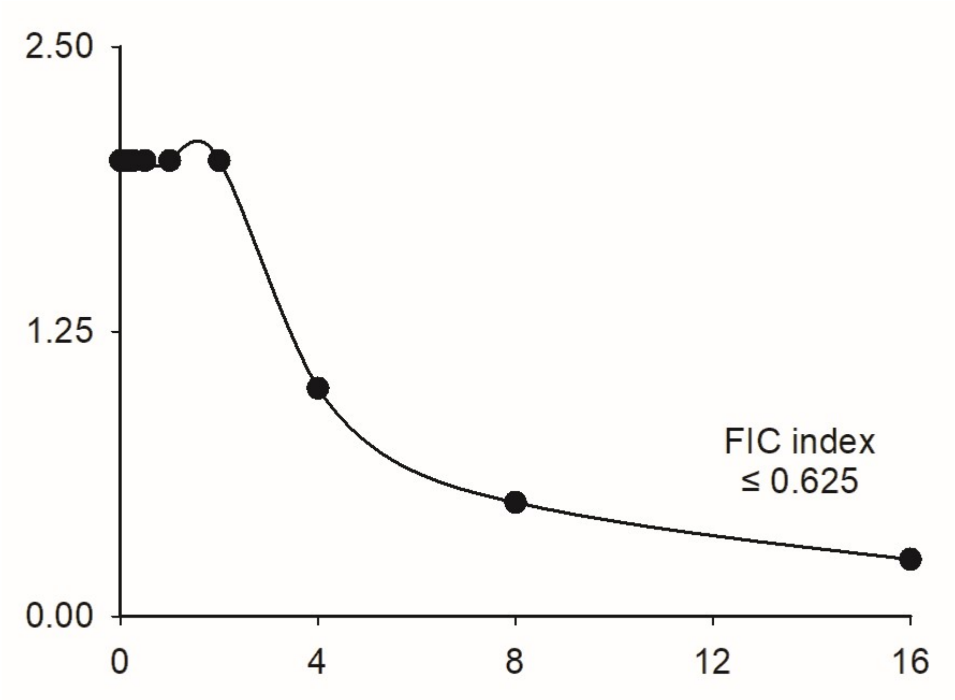

Figure S3c

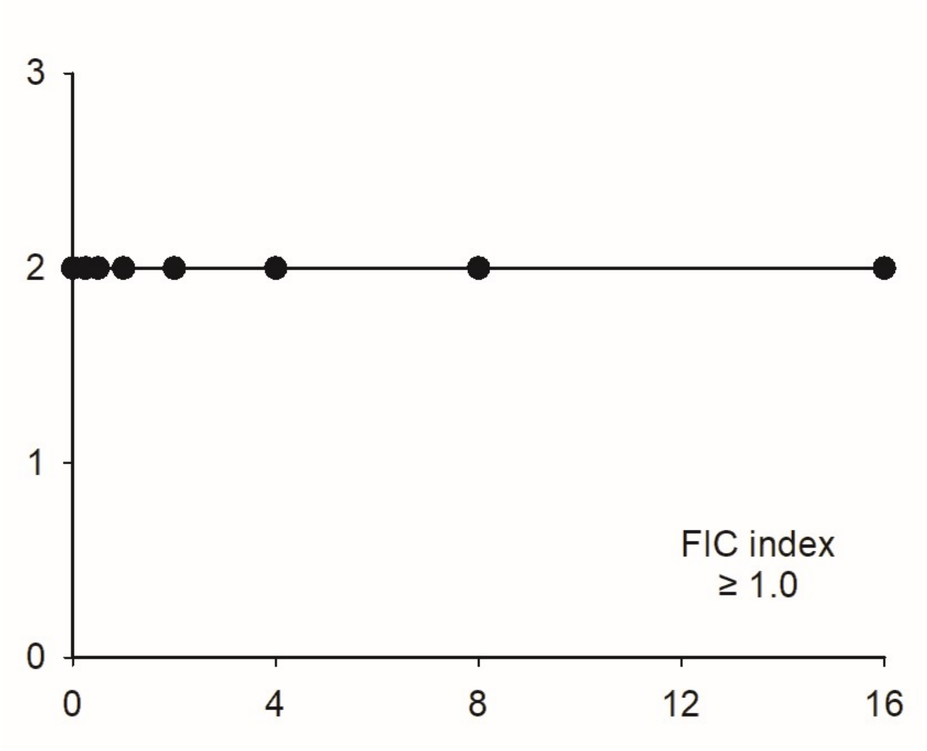

Figure S3d

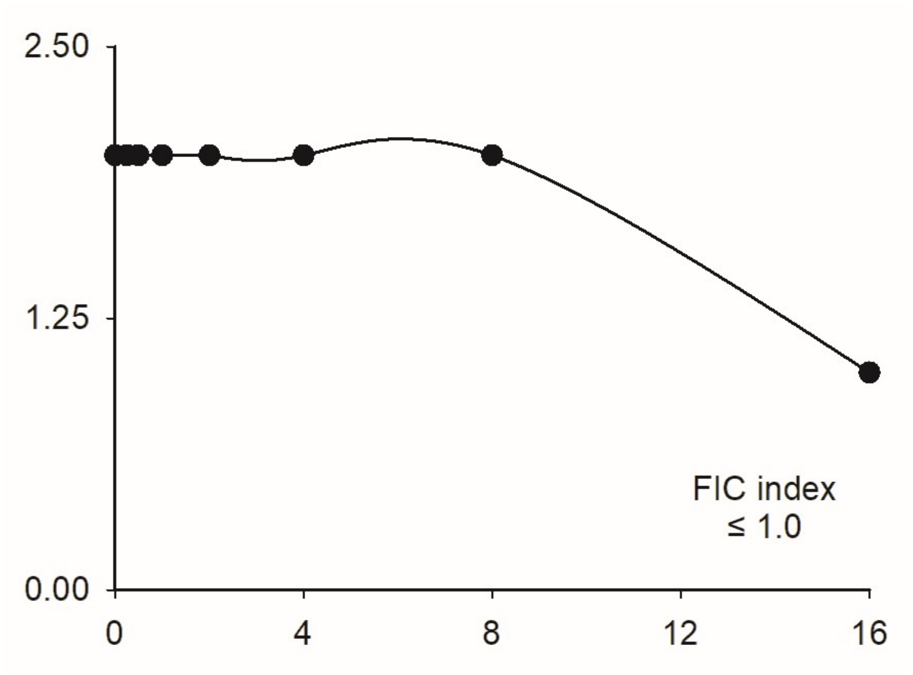

Supplementary Figure S3. Modulation of ciprofloxacin and tetracycline antibacterial activity by xanthines (caffeine and pentoxifylline) in *Enterococcus faecium* using microbroth dilution assay and checkerboard methodology. (**a**), ciprofloxacin-caffeine mixtures; (**b**), tetracycline-caffeine mixtures; (**c**), ciprofloxacin-pentoxifylline mixtures; (**d**), tetracycline-pentoxifylline mixtures. FIC, Fractional Inhibitory Concentration Index calculated for each tested antibiotic-xanthine combination according to Odds [33]

Figure S4a

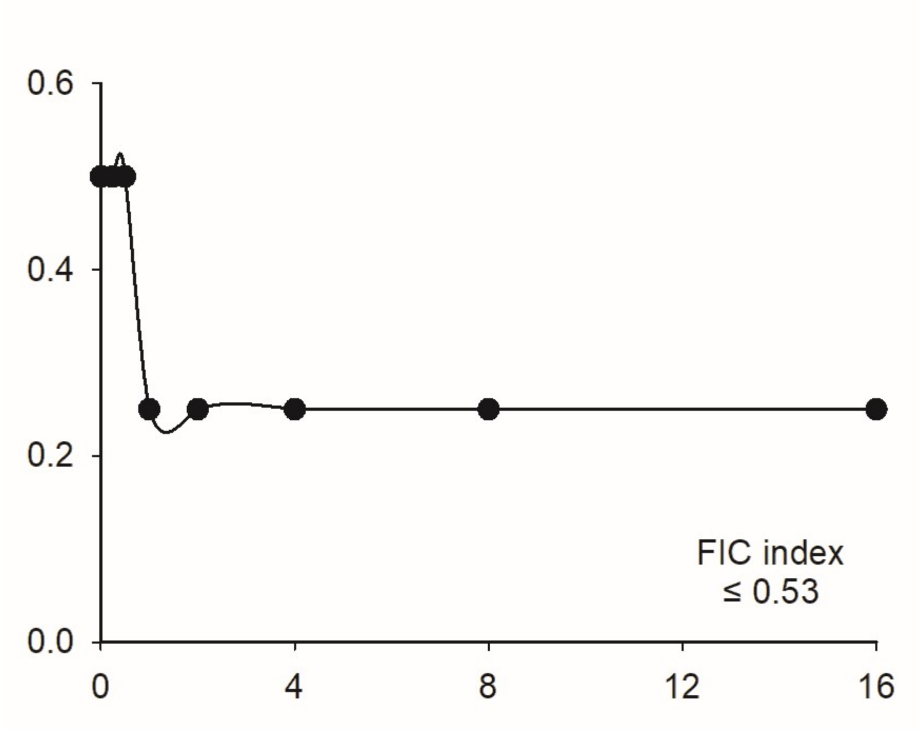

Figure S4b

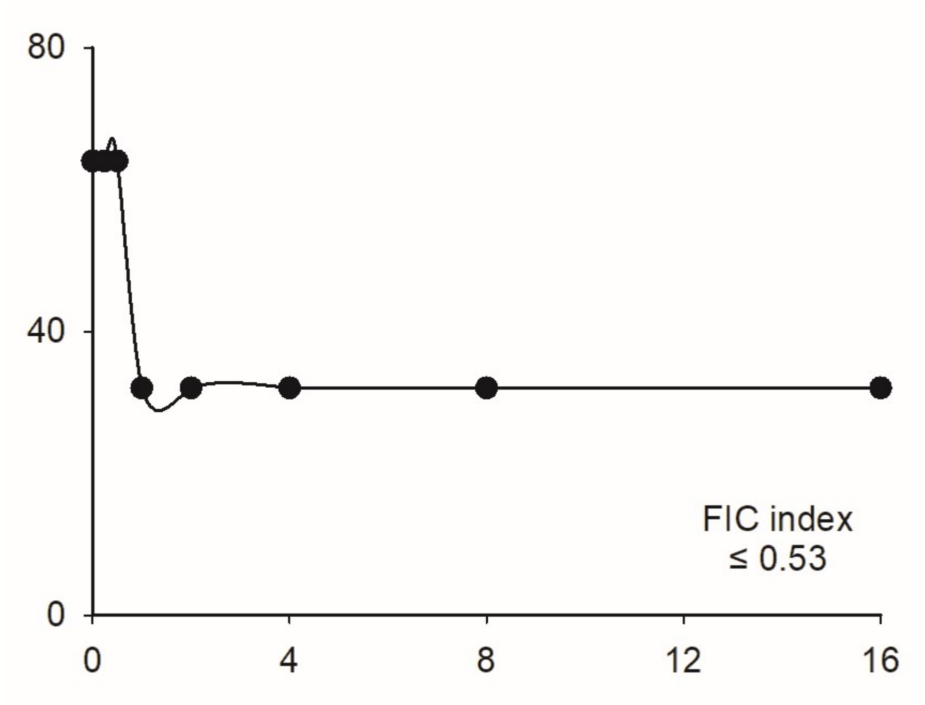

Figure S4c

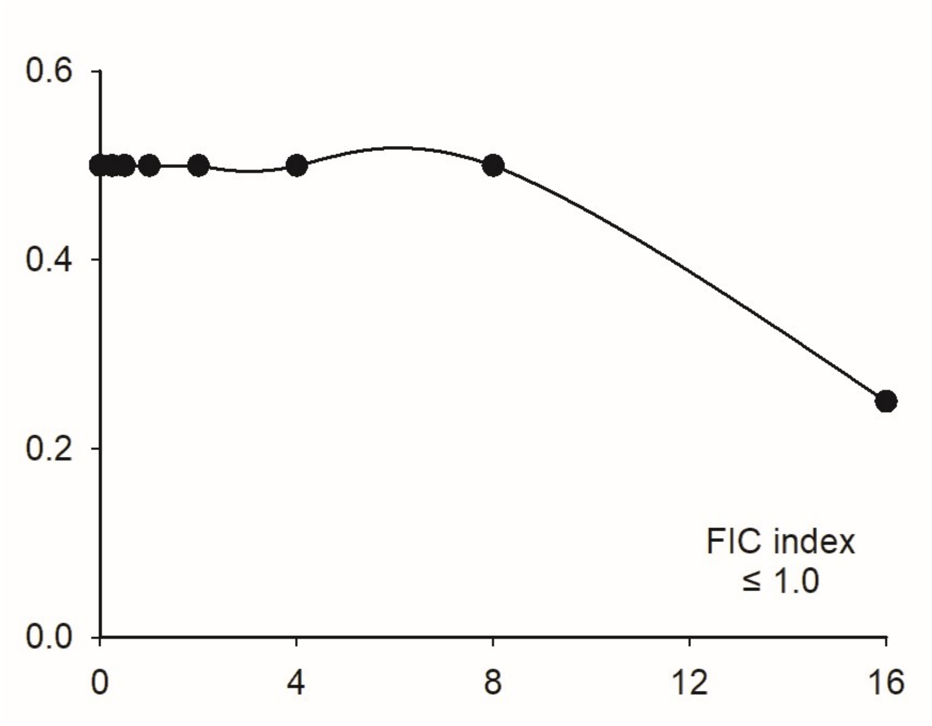

Figure S4d

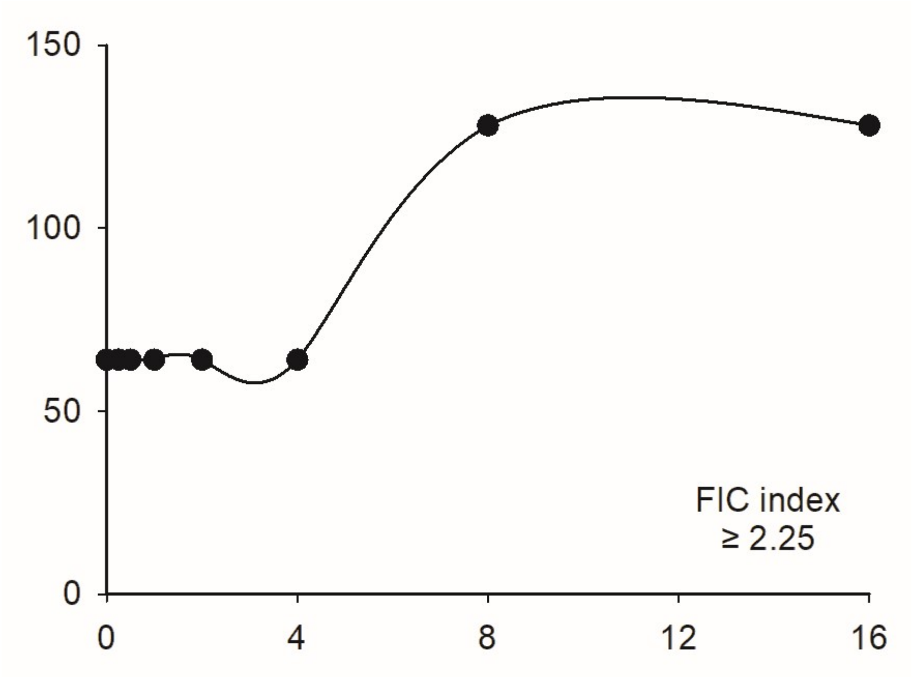

Supplementary Figure S4. Modulation of ciprofloxacin and tetracycline antibacterial activity by xanthines (caffeine and pentoxifylline) in *Pseudomonas aeruginosa* using microbroth dilution assay and checkerboard methodology. (**a**), ciprofloxacin-caffeine mixtures; (**b**), tetracycline-caffeine mixtures; (**c**), ciprofloxacin-pentoxifylline mixtures; (**d**), tetracycline-pentoxifylline mixtures. FIC, Fractional Inhibitory Concentration Index calculated for each tested antibiotic-xanthine combination according to Odds [33]

Figure S5a

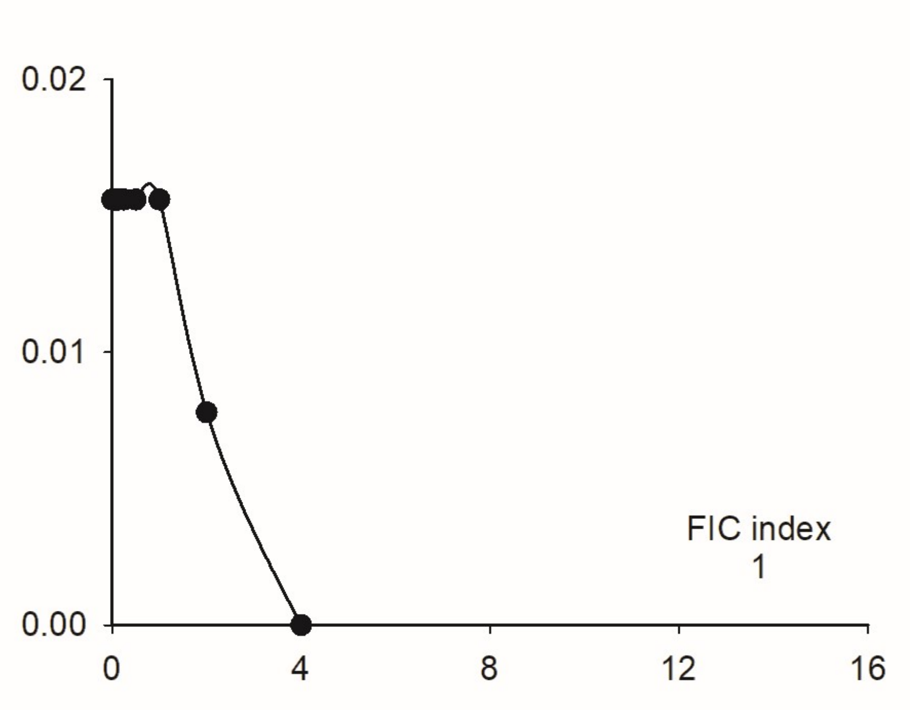

Figure S5b

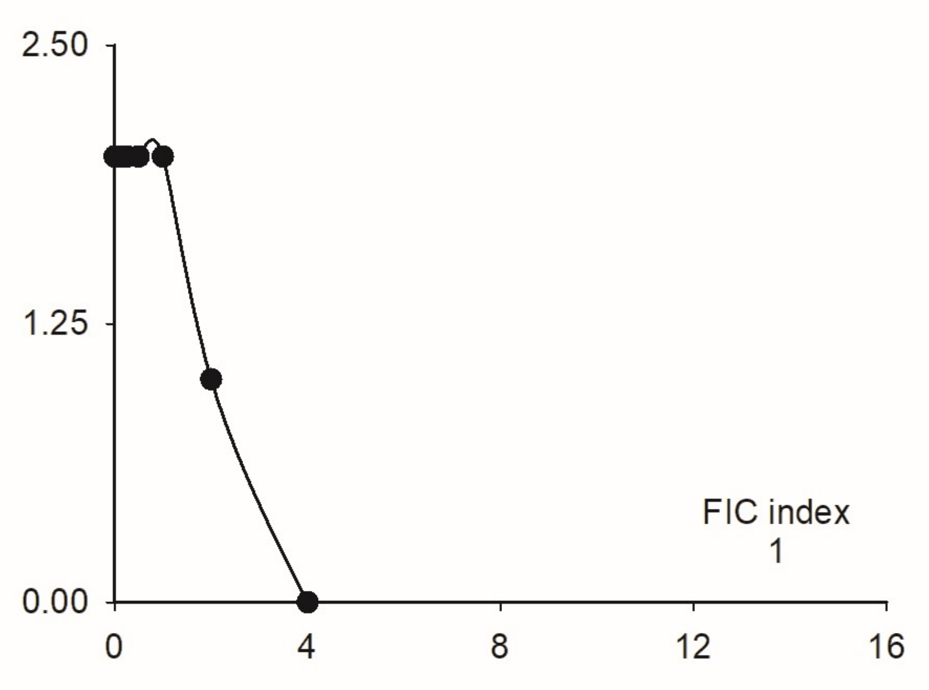

Figure S5c

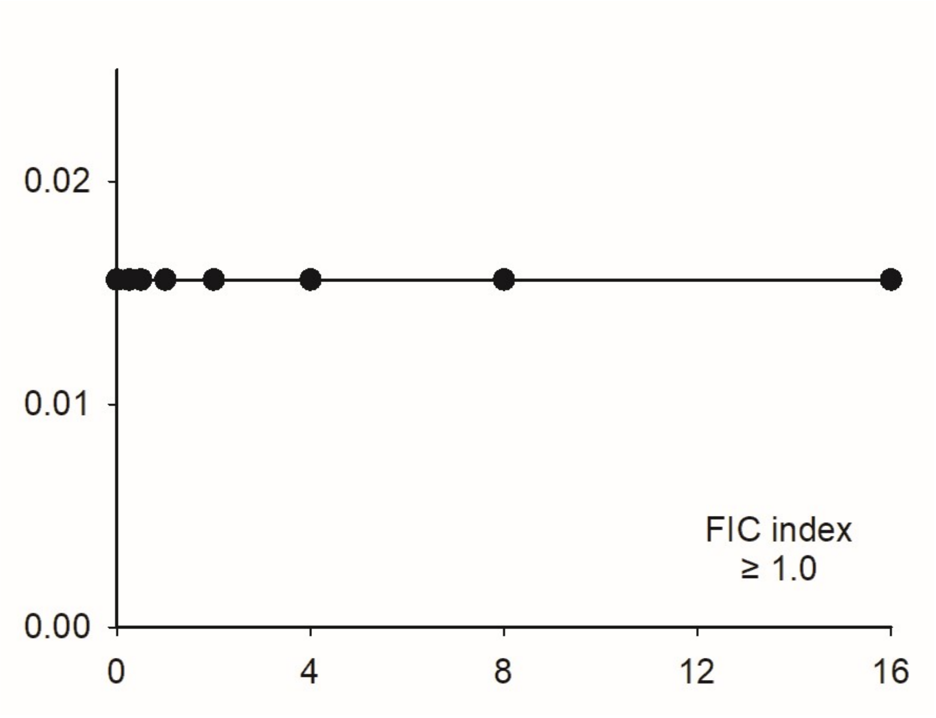

Figure S5d

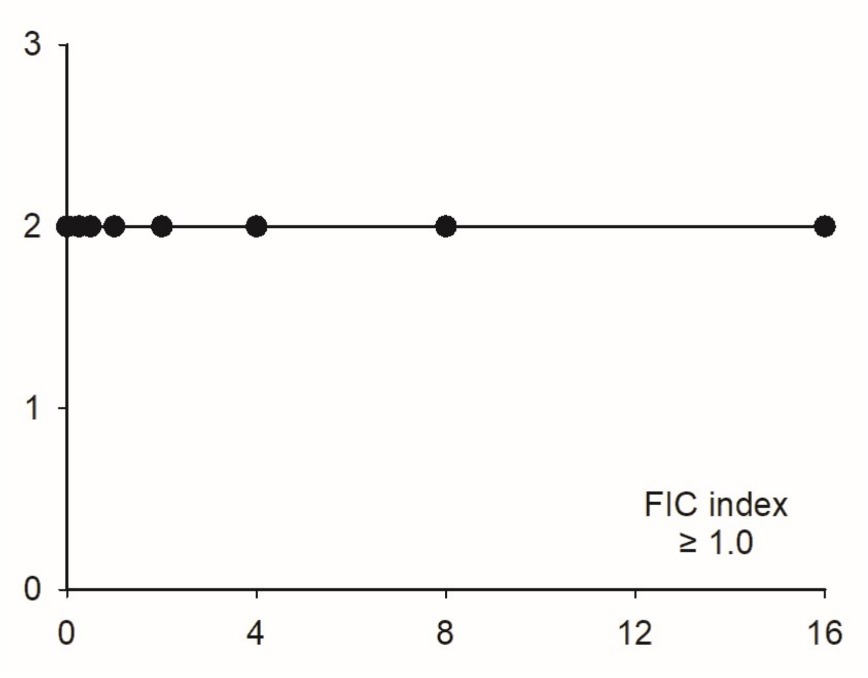

Supplementary Figure S5. Modulation of ciprofloxacin and tetracycline antibacterial activity by xanthines (caffeine and pentoxifylline) in *Escherichia coli* using microbroth dilution assay and checkerboard methodology. (**a**), ciprofloxacin-caffeine mixtures; (**b**), tetracycline-caffeine mixtures; (**c**), ciprofloxacin-pentoxifylline mixtures; (**d**), tetracycline-pentoxifylline mixtures. FIC, Fractional Inhibitory Concentration Index calculated for each tested antibiotic-xanthine combination according to Odds [33]

Figure S6a

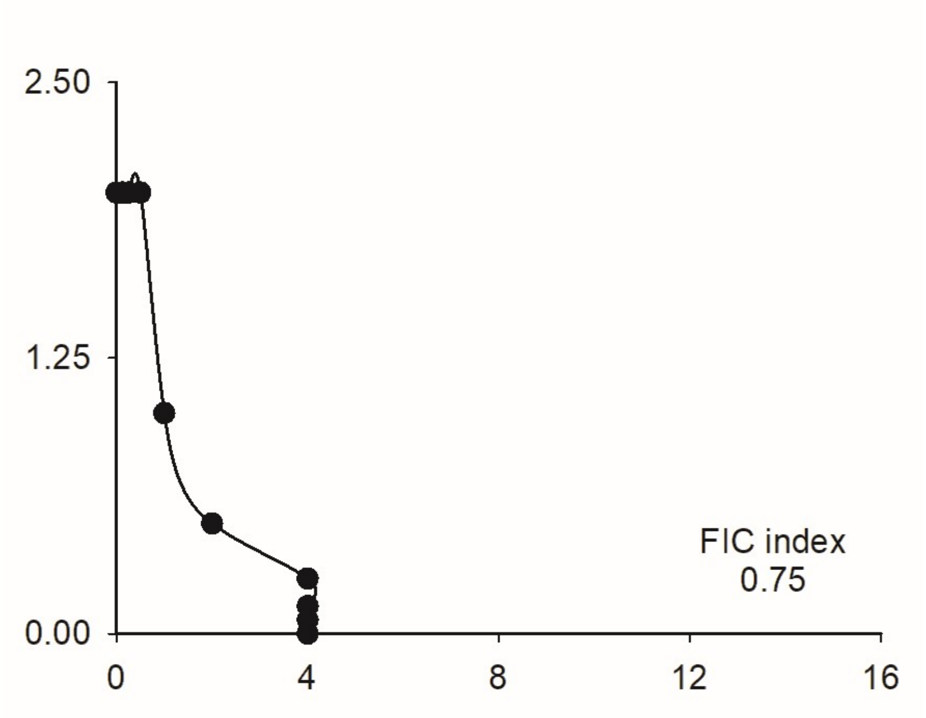

Figure S6b

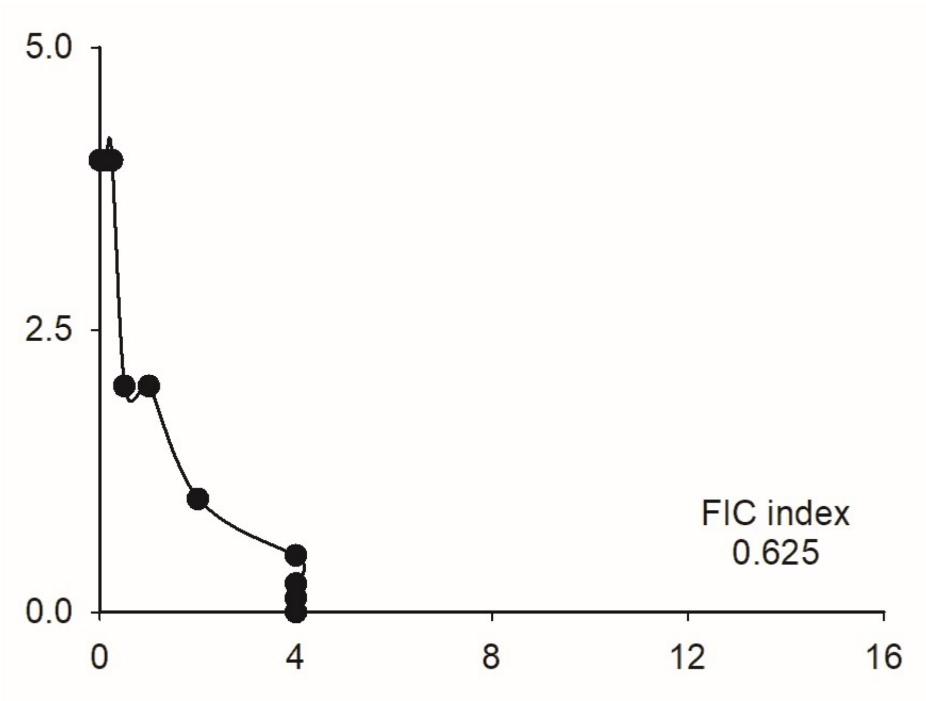

Figure S6c

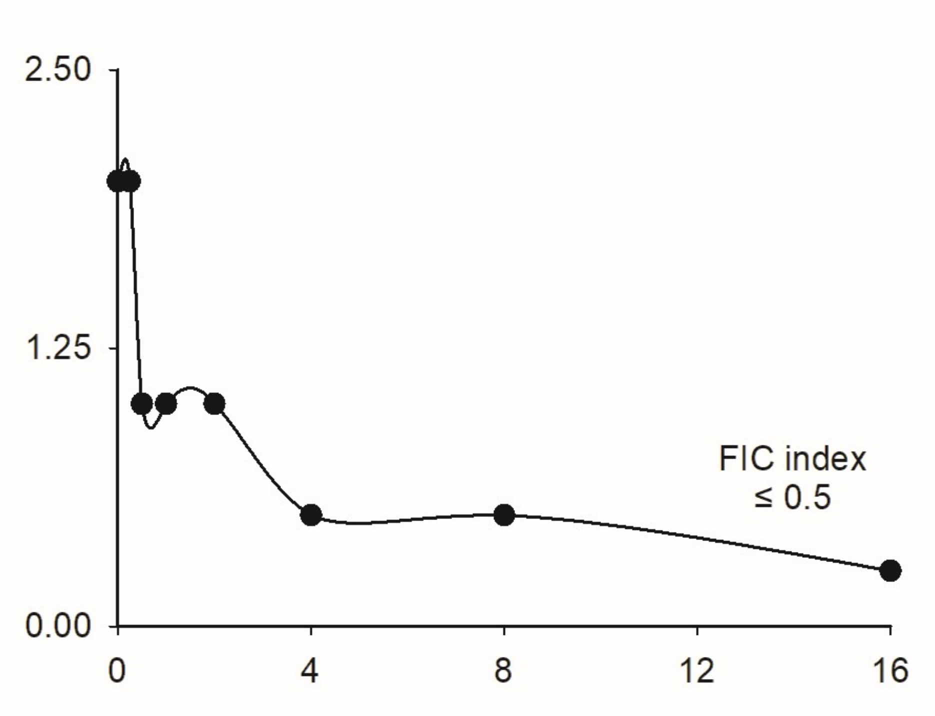

Figure S6d

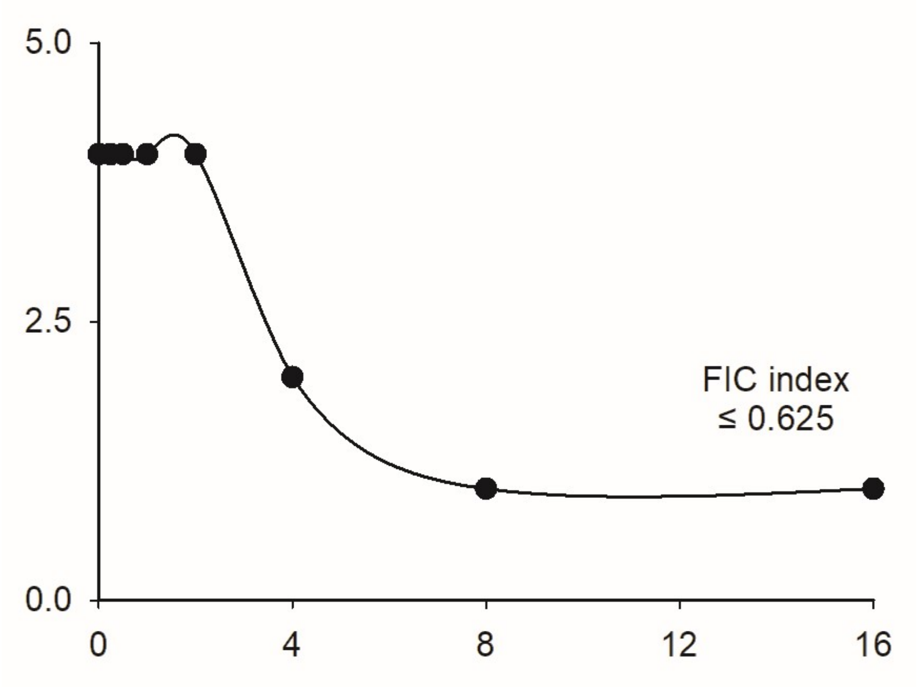

Supplementary Figure S6. Modulation of ciprofloxacin and tetracycline antibacterial activity by xanthines (caffeine and pentoxifylline) in *Acinetobacter baumannii* using microbroth dilution assay and checkerboard methodology. (**a**), ciprofloxacin-caffeine mixtures; (**b**), tetracycline-caffeine mixtures; (**c**), ciprofloxacin-pentoxifylline mixtures; (**d**), tetracycline-pentoxifylline mixtures. FIC, Fractional Inhibitory Concentration Index calculated for each tested antibiotic-xanthine combination according to Odds [33]

Figure S7a

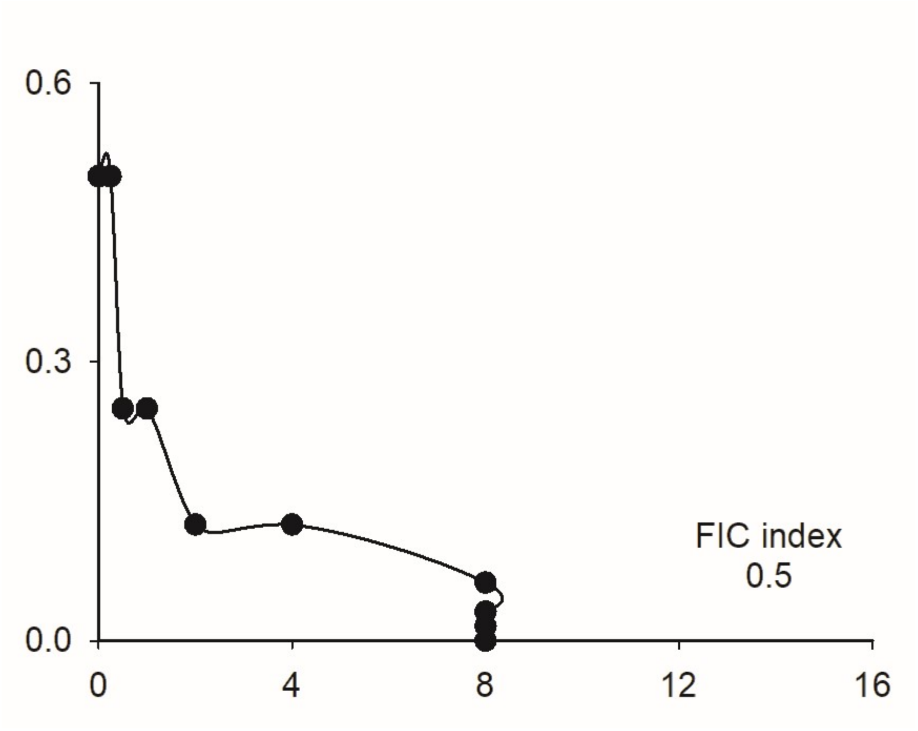

Figure S7b

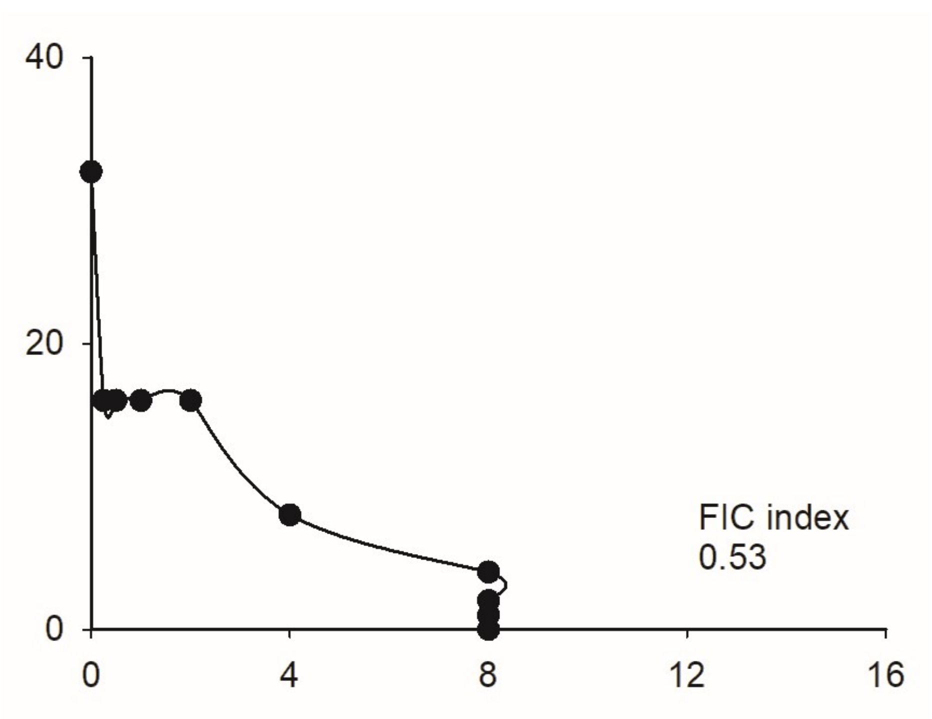

Figure S7c

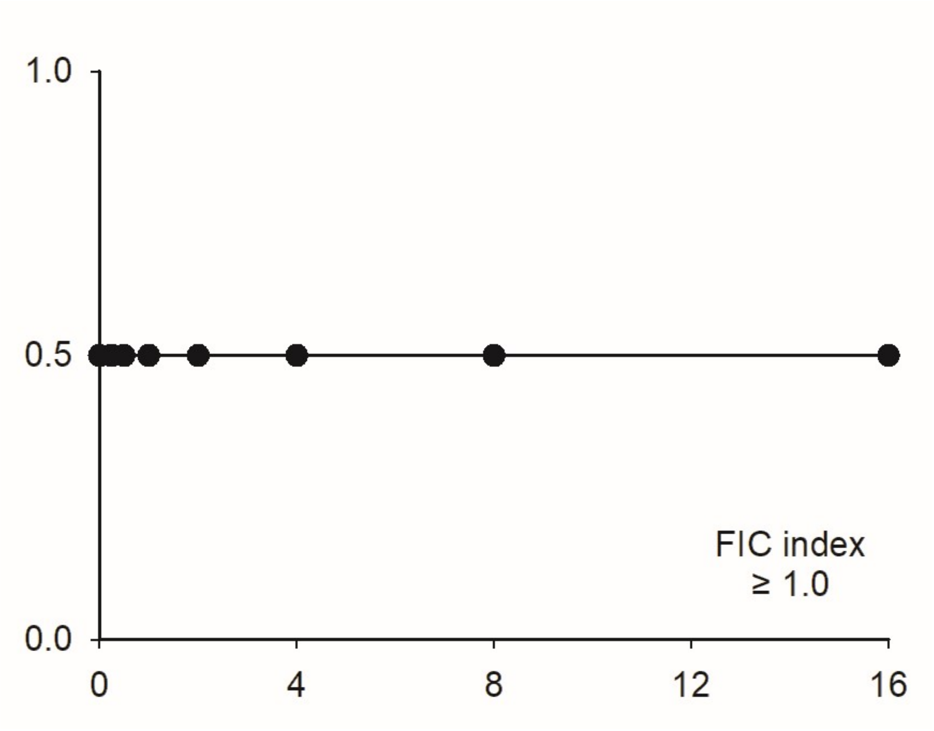

Figure S7d

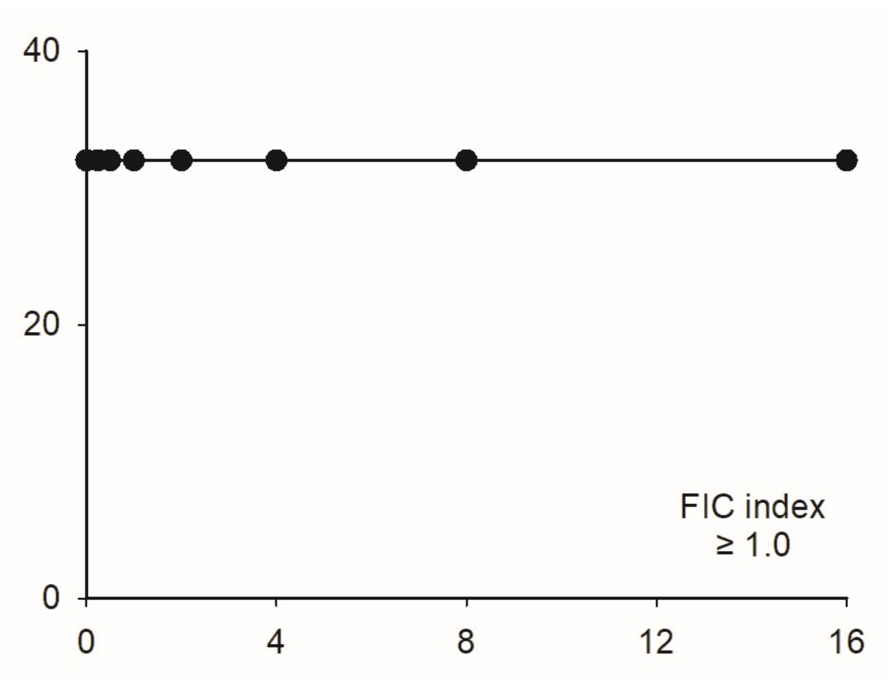

Supplementary Figure S7. Modulation of ciprofloxacin and tetracycline antibacterial activity by xanthines (caffeine and pentoxifylline) in *Klebsiella pneumoniae* using microbroth dilution assay and checkerboard methodology. (**a**), ciprofloxacin-caffeine mixtures; (**b**), tetracycline-caffeine mixtures; (**c**), ciprofloxacin-pentoxifylline mixtures; (**d**), tetracycline-pentoxifylline mixtures. FIC, Fractional Inhibitory Concentration Index calculated for each tested antibiotic-xanthine combination according to Odds [33]

Figure S8a

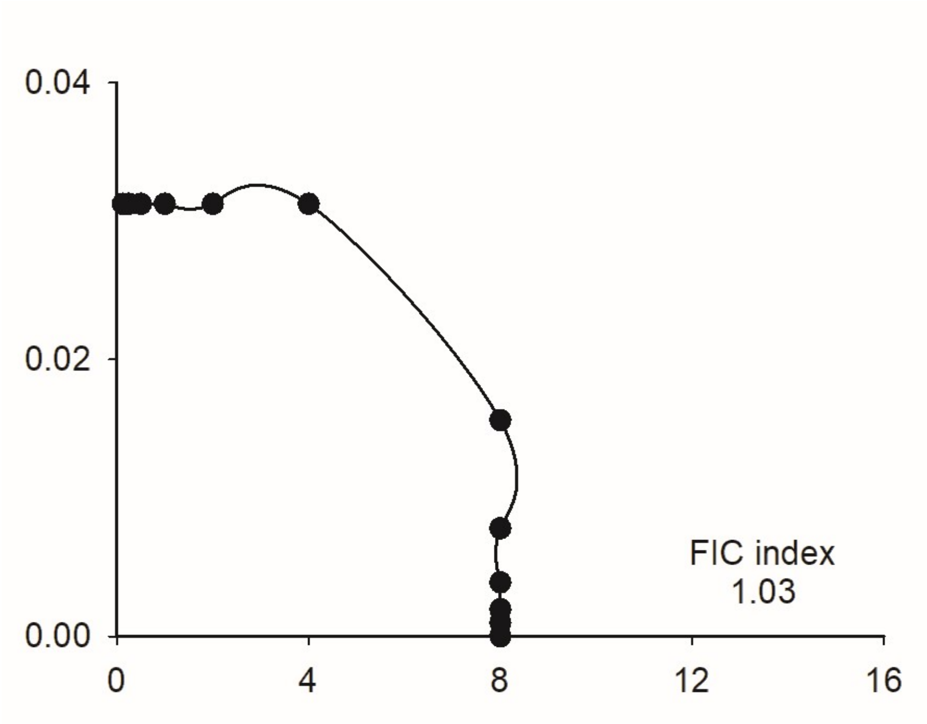

Figure S8b

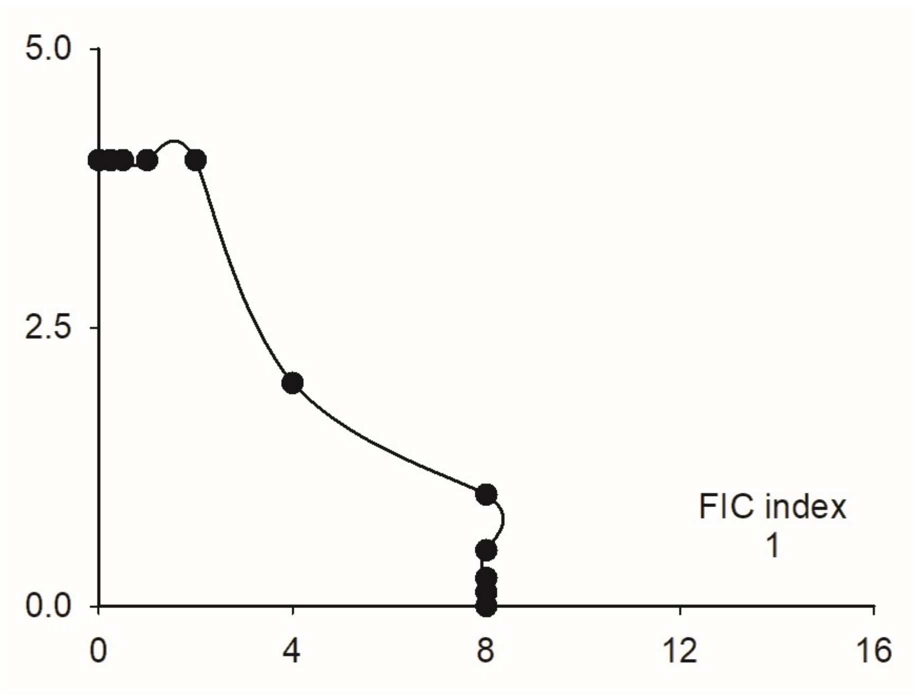

Figure S8c

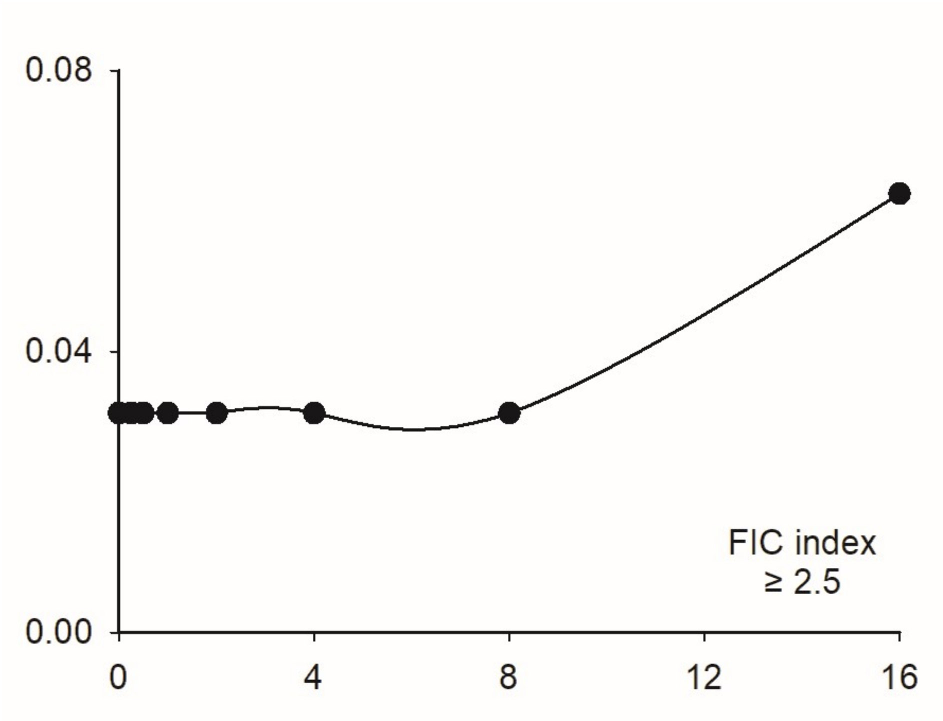

Figure S8d

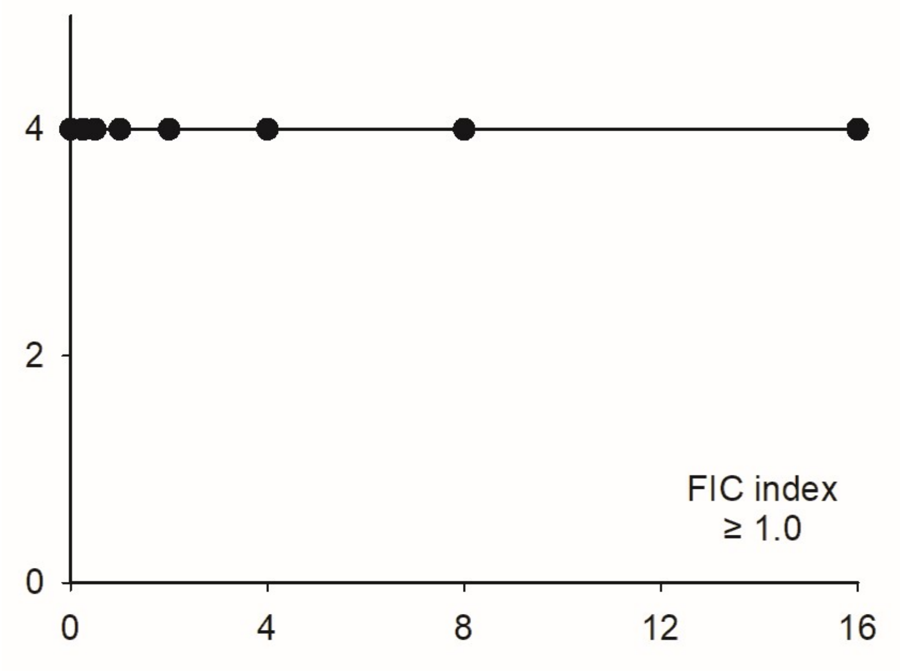

Supplementary Figure S8. Modulation of ciprofloxacin and tetracycline antibacterial activity by xanthines (caffeine and pentoxifylline) in *Enterobacter cloacae* using microbroth dilution assay and checkerboard methodology. (**a**), ciprofloxacin-caffeine mixtures; (**b**), tetracycline-caffeine mixtures; (**c**), ciprofloxacin-pentoxifylline mixtures; (**d**), tetracycline-pentoxifylline mixtures. FIC, Fractional Inhibitory Concentration Index calculated for each tested antibiotic-xanthine combination according to Odds [33]
